# An Overview of Bioactive 1,3-Oxazole-Containing Alkaloids from Marine Organisms

**DOI:** 10.3390/ph14121274

**Published:** 2021-12-06

**Authors:** Jinyun Chen, Sunyan Lv, Jia Liu, Yanlei Yu, Hong Wang, Huawei Zhang

**Affiliations:** 1 School of Pharmaceutical Sciences, Zhejiang University of Technology, Hangzhou 310014, China; chenjinyun07@163.com (J.C.); lvsunyan@163.com (S.L.); jliu3092@163.com (J.L.); hongw@zjut.edu.cn (H.W.); 2 Collaborative Innovation Center of Green Pharmaceutics of Delta Yangzi Region, Zhejiang University of Technology, Hangzhou 310014, China; yanleiyu@zjut.edu.cn; 3 Key Laboratory of Marine Fishery Resources Exploitment & Utilization of Zhejiang Province, Hangzhou 310014, China

**Keywords:** 1,3-oxazole, alkaloid, marine natural product, bioactive property, marine organism, marine sponge, symbiotic microorganism

## Abstract

1,3-Oxazole chemicals are a unique class of five-membered monocyclic heteroarenes, containing a nitrogen atom and an oxygen. These alkaloids have attracted extensive attention from medicinal chemists and pharmacologists owing to their diverse arrays of chemical structures and biological activities, and a series of 1,3-oxazole derivatives has been developed into therapeutic agents (e.g., almoxatone, befloxatone, cabotegravir, delpazolid, fenpipalone, haloxazolam, inavolisib). A growing amount of evidence indicates that marine organisms are one of important sources of 1,3-oxazole-containing alkaloids. To improve our knowledge regarding these marine-derived substances, as many as 285 compounds are summarized in this review, which, for the first time, highlights their sources, structural features and biological properties, as well as their biosynthesis and chemical synthesis. Perspective for the future discovery of new 1,3-oxazole compounds from marine organisms is also provided.

## 1. Introduction

**Figure 1:**
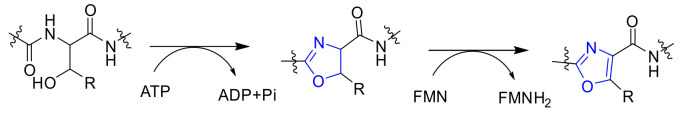
Proposed biosynthetic pathway for 1,3-oxazoles.

Natural products and their structural analogs are an invaluable source of inspiration in drug design and development. 1,3-Oxazole chemicals are a unique class of five-membered monocyclic heteroarenes, containing a nitrogen atom and an oxygen atom, and usually possess a 2,4- or 2,5-substitution pattern with diverse chemical structures. It is noteworthy that these alkaloids display a wide range of biological properties, such as antibacterial, antifungal, anti-inflammatory, antimalarial, antioxidant, antiviral, cytotoxic, insecticidal, repellent activity, and an inhibitory effect on kinase, showcasing great therapeutic potential [1,2]. A growing amount of evidence indicates that 1,3-oxazole heterocycle is a fundamental structural motif found in marine natural products, which is putatively biosynthesized by the cyclodehydration and dehydrogenation of two amino acids (Figure 1) [3].

**Figure 2:**
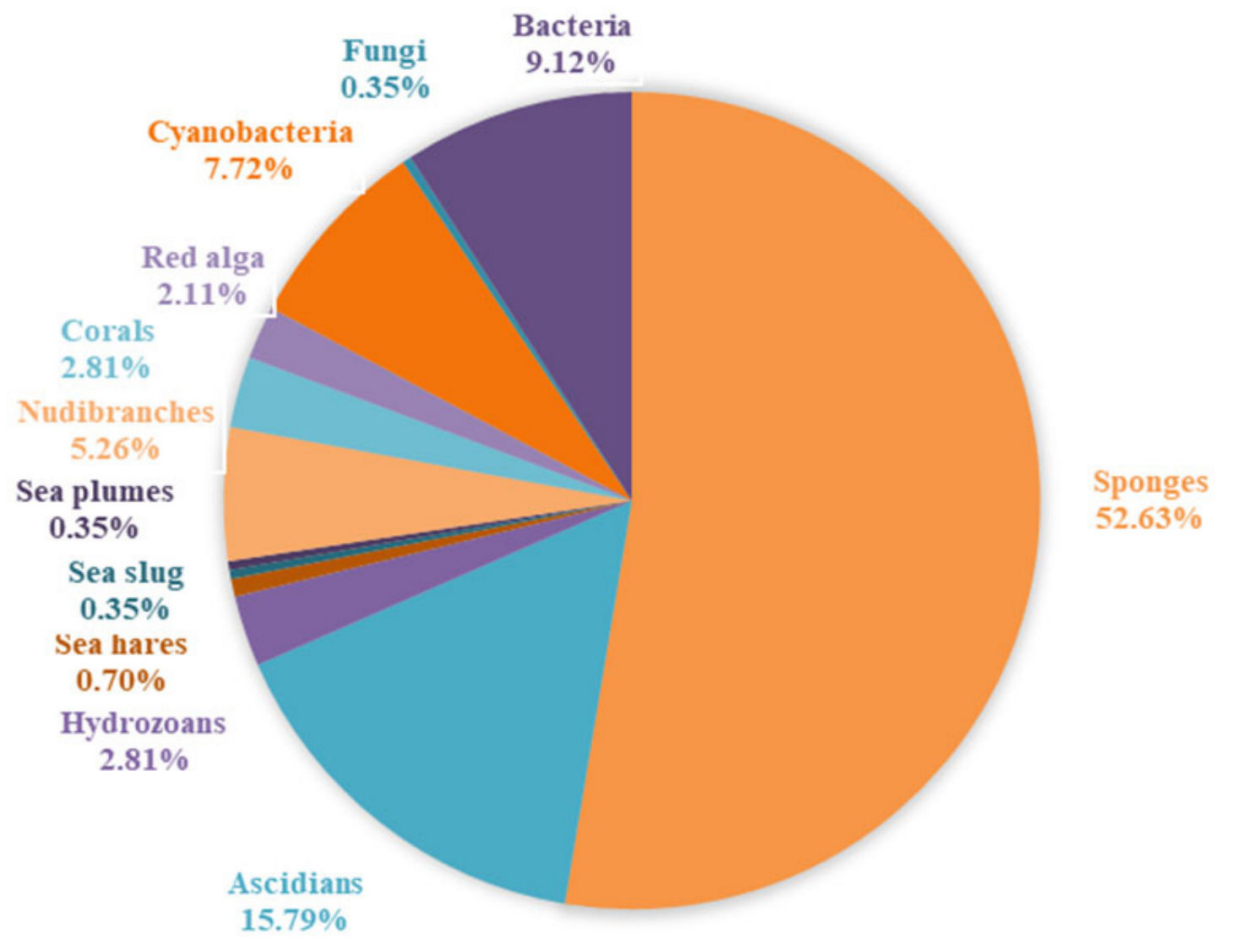
Percentage distribution of 1,3-oxazole-containing alkaloids from marine organisms.

By an extensive and in-depth literature search using DNP (dictionary of natural products) and SciFinder tools, a total of 285 marine-derived 1,3-oxazole-containing compounds (**1**–**285**) were isolated and reported until 2020. Almost 80% of these substances were produced by animals (sponges, ascidian, nudibranch, hydrozoan, coral, sea hare and plume) followed by plants (cyanobacteria and red algae) and microorganisms (bacteria and fungi) (Figure 2). To better understand these marine-derived 1,3-oxazole substances and promote marine drugs, here, a systematic and comprehensive review is summarized for the first time. It highlights their biological sources, structural features and bioactivities, as well as biosynthesis and chemical synthesis by comparing with two recent review works, in which one mainly focuses on the biological activities of synthetic and natural oxazoles [4], and the other puts more emphasis on the sources, biological activities, structural features and chemical synthesis of natural oxazole-containing peptides [5]. On the basis of chemical structures, these 1,3-oxazole-based alkaloids are grouped into four major types including peptide, macrolide, polyketide and benzoxazole, and are respectively introduced herein. Detailed information for these natural products is supplied in the Supplementary Materials.

## 2. Peptides

### 2.1. Linear Peptides

**Scheme 1:**
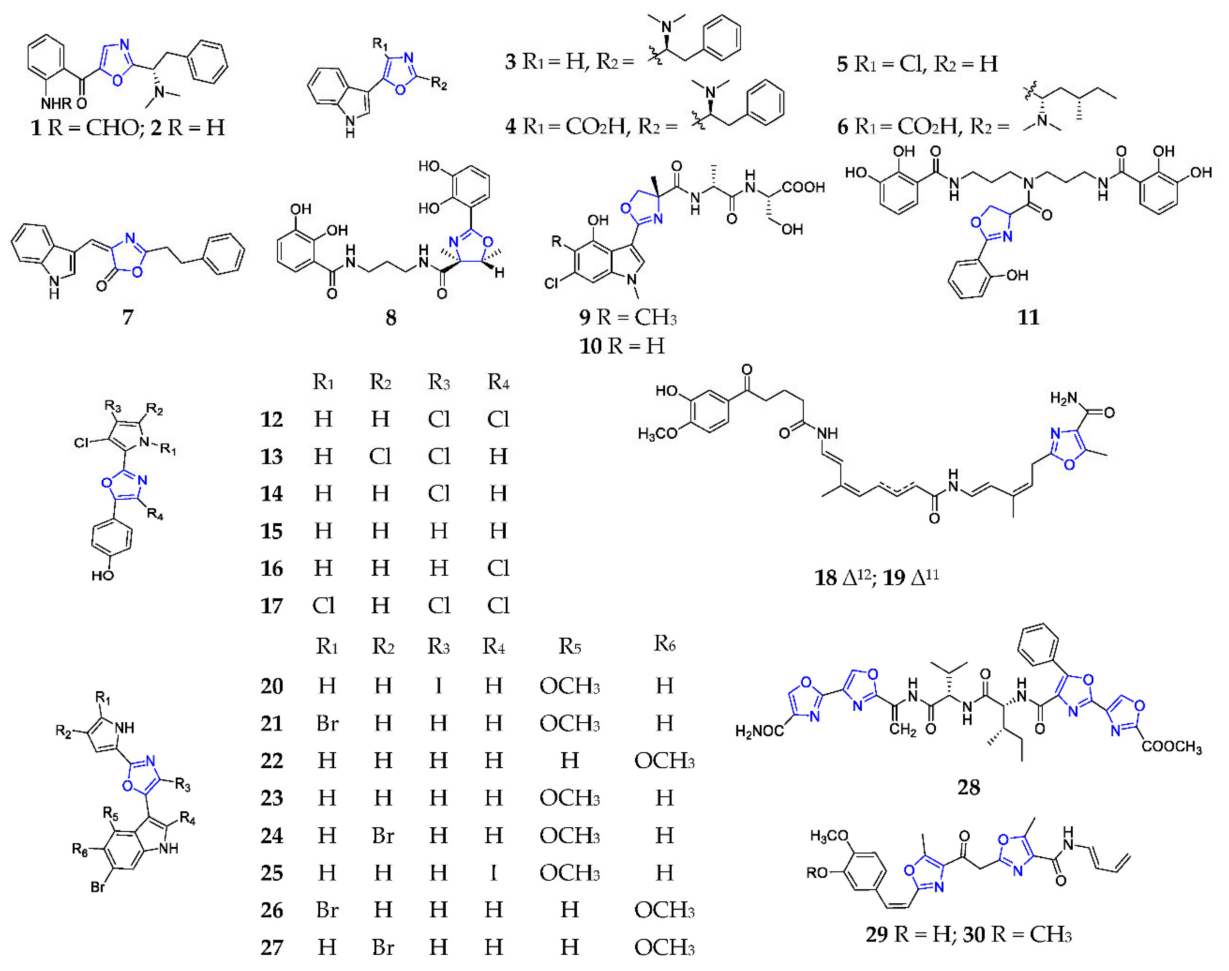
Marine-derived 1,3-oxazole-containing linear peptides (**1**–**30**).

Marine algae and microorganisms are the major sources of linear peptides with the 1,3-oxazole motif. The chemical investigation of a red seaweed of *Delesseriaceae* from the coasts of north Dakar (Dakar, Senegal) successively led to the isolation of four new linear dipeptides almazoles A–D (**1**–**4**) (Scheme 1), in which each compound bears an unusual 2,5-disubstituted oxazole moiety [6,7,8]. Absolute structures of these algal metabolites were originally determined by biomimetic synthesis from *L*-tryptophan with *N*,*N*-dimethyl-*L*-phenylalanin, for which Robinson–Gabriel cyclization served as the key oxazole forming step [6]. Lately, almazole C (**3**) has been well prepared by a four-step synthesis, with a 32% overall yield through the Aza-Wittig reaction [9], while compound **4** was the first bioactive member of the almazole family, possessing potent antibacterial against *Serratia marcescens* and *Salmonella typhi* XLD [8], and its original structure was revised as 5-(3-indolyl)oxazole-4-carboxylic acid by Horne and his coworkers [10]. Streptochlorin (**5**) and martefragin A (**6**) are another two novel 5-(3-indolyl)oxazole-containing alkaloids, respectively produced by marine strain *Streptomyces* sp. 04DH110 from the east sea of Korea [11] and the red algae *Martensia fragilis* collected off the coast of Uozu (Uozu, Japan) [12]. In addition to potent fungicidal activity against *Pythium*, *Botrytis cinerea*, *Septoria tritici*, *Pyricularia Oryzae*, *Fusarium culmorum* and *Rhizoctonia solani*, compound **5** displayed a strong inhibitory effect on angiogenesis by blocking NF-κB signaling, as well as human leukemia and normal liver cell lines with IC_50_ values of 1.05 and 10.9 µg/mL, respectively [11,13,14]. It is noteworthy that one three-step synthesis of compound **5** had been readily achieved by sequential asmic-ester condensations and sulfanyl-Lithium exchange-trapping [15]. As a promising inhibitor of lipid peroxidation, compound **6** was firstly synthesized by Nishida and coworkers in 1998, and its absolute configuration was unambiguously determined as ′′*S*, ′′*S* [16]. Almazolone (**7**) had similar biogenetic features to compounds **1**–**4** and was purified from Senegal red alga *Haraldiophyllum* sp. [17].

Co-culture of *Serratia* sp. and *Shewanella* sp. resulted in the production of serratiochelin A (**8**), which displayed antiproliferative activity against human melanoma cell lines and non-malignant lung fibroblasts, as well as antimicrobial activity toward *Staphylococcus aureus* [18]. Two new indole-containing peptides, JBIR-34 (**9**) and JBIR-35 (**10**), were produced by one symbiotic strain *Streptomyces* sp. Sp080513GE-23 from *Haliclona* sp., and exhibited a potent DPPH radical scavenging capability with IC_50_ values of 1.0 and 2.5 mM, respectively [19]. The unique 4-methyloxazoline moiety was formed by a α-methyl-*L*-serine from *D*-alanine and a 5,10-methylene-tetrahydrofolate by FmoH [20]. Nigribactin (**11**) was isolated as a new siderophore from marine *Vibrio nigripulchritudo* and was shown to enhance the expression of the *spa* encoding protein A [21]. Phorbazoles A–D (**12**–**15**) were a novel class of marine alkaloids, containing chlorinated pyrrole and 1,3-oxazole moieties from the Indo-Pacific sponge *Phorbas aff. clathrata* (Levi) [22]. Recently, the chemical preparation of phorbazole B (**13**) has been achieved by simple catalytic chlorination and iodization to protect the oxazole ring [23]. The first chemical analysis of the marine mollusc *Aldisa andersoni,* collected off Muttom coast, (Kanyakumari, India) afforded two new cytotoxic phorbazole analogs, 9-chloro-phorbazole D (**16**) and *N*1-methyl-phorbazole A (**17**), as well as phorbazoles A (**2**), B (**13**) and D (**15**) [24].

Two novel linear and achiral polyketide-peptides, ariakemicins A (**18**) and B (**19**), were obtained as an inseparable mixture from one marine gliding bacterium *Rapidithrix* sp., collected off the muddy land alongside the Ariake Inland Sea, and displayed selectively antimicrobial activities against Gram-positive bacteria (*Brevibacterium* sp., *S. aureus*, and *Bacillus subtilis*), and weak cytotoxicity against cancer cell lines A549 and BHK [25]. Breitfussins A–H (**20**–**27**) were the first marine natural products containing an indole-oxazole-pyrrole framework from hydrozoan *Thuiaria breitfussi* inhabits in the Arctic ocean, and were found to excellently inhibit PIM1 and DRAK1 kinases [26,27]. Furthermore, breitfussin C (**22**) strongly exhibited a cytotoxic effect on cancer cell lines (MCF-7, HT-29, MOLT-4, MV-4-11 and MRC-5). One concise total synthesis of the halogen-rich dipeptides **20** and **21** was created by Bayer and his coworkers in 2015, which consists of the palladium-catalyzed cross-coupling of indole and pyrrole on an oxazole core and selective lithiation/iodination of a common indole-oxazole fragment [28]. Mechercharmycin B (**28**) was a new linear peptide containing four 1,3-oxazole rings produced by the marine strain *Thermoactinomyces* sp. YM3-251 from Mecherchar (Palau) [29]. Siphonazole (**29**) and dimethoxy analog (**30**) were the first naturally occurring substances that incorporated oxazole subunits connected by a two-carbon tether from a marine microbe *Herpetosiphon* sp., and exhibited selective cytotoxicity to human breast cancer HTB-129 and acute T cell leukemia TIB-152 [30]. The first chemical synthesis of **29** was achieved in 2007 through the preparation of an oxazole ring using rhodium carbene, and the installation of the pentadienyl amino side-chain [31].

### 2.2. Monocyclic Peptides

#### 2.2.1. Pentapeptides

**Scheme 2:**
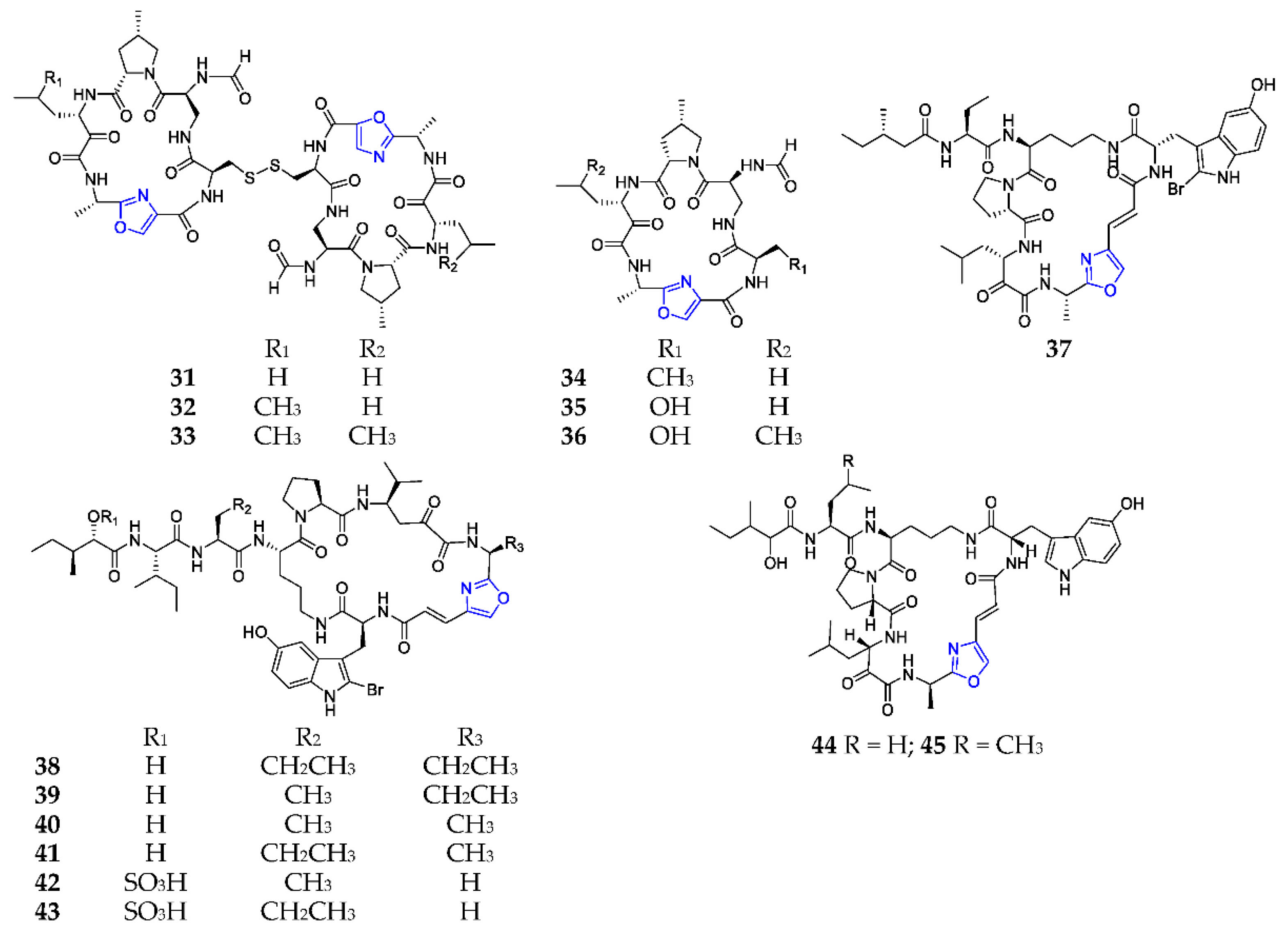
Marine-derived 1,3-oxazole-containing monocyclic pentapeptides (**31**–**45**).

To the best of our knowledge, the marine sponge genus *Theonella* is the most common and important producer of 1,3-oxazole-containing pentapeptides. An inseparable mixture of three dimeric cyclic pentapeptides, nazumazoles A–C (**31**–**33**) (Scheme 2), was obtained from the aqueous alcoholic extract of *Theonella swinhoei,* collected from Hachijo Island (Tokyo, Japan), and exhibited a potent cytotoxicity against P388 murine leukemia cells with an IC_50_ value of 0.83 μM [32]. Subsequently, three new monomeric analogs, nazumazoles D–F (**34**–**36**), were purified from the same specimen and shown to remarkably inhibit chymotrypsin with IC_50_ values of 2, 3 and 10 μM, respectively [33]. All absolute configurations of the amino acid residues in compounds **31**–**36** were unambiguously determined by Marfey’s method. Seven 2-bromotryptophan-containing pentapeptides, orbiculamide A (**37**), keramamides B–E (**38**–**41**), M (**42**), and N (**43**) from an Okinawan *Theonella* sp., displayed, in vitro, a strongly cytotoxic effect on cell lines P388, LI210 and KB [34]. Discobahamins A (**44**) and B (**45**) were isolated from a deep-water marine sponge *Discodermia* sp. collected off Goulding Cay (Goulding Cay, Bahamas), and were shown to have weak antifungal activity against *Candida albicans* [35].

#### 2.2.2. Hexapeptides

**Scheme 3:**
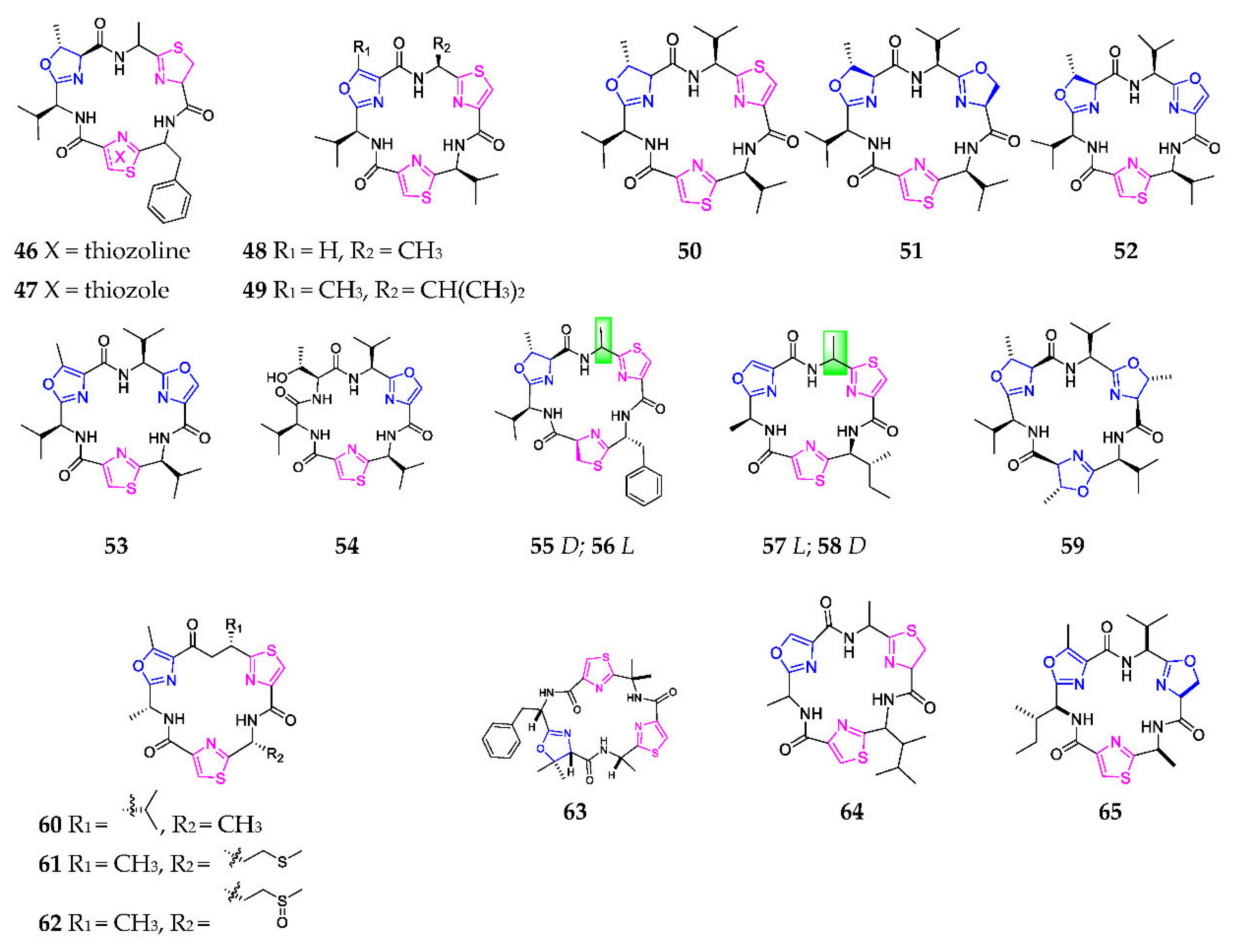
Marine-derived 1,3-oxazole-containing monocyclic hexapeptides (**46**–**65**).

Ascidians of the genera *Lissoclinum* and *Didemnum* are prolific producers of cyclic hexapeptides with 1,3-oxazole moieties and thiazole, thiazoline or oxazoline rings. During the period from 1989 to 2017, a total of thirteen hexapeptides, bistratamides A–N (**46**–**58**) (Scheme 3), had been found in ascidian *L. bistratum*. The structure-activity relationship (SAR) analysis indicated that bistratamides containing two thiazole rings possessed stronger cytotoxic activity than others containing one thiazole ring and/or one oxazole ring [36,37,38,39,40]. Westiellamide (**59**) and dendroamides A-C (**60**–**62**) are new bistratamide analogs derived from blue-green algae. Compound **59** exhibited moderate cytotoxicity against KB and LoVo cell lines at 2 μg/mL and **60** showed excellent activity toward multidrug resistant tumors [41,42]. Chemical study of the extracts of ascidian *Didemnum molle* collected in Madagascar afforded didmolamide A (**63**), with a mild cytotoxic effect on tumor cell lines A549, HT29 and MEL28 [43]. Bertram and his coworkers firstly invented a novel method to synthesize bistratamides and didmolamides by mediating the coupling reaction of azole amino acids via FDPP-i-Pr2NEt [44].

**Scheme 4:**
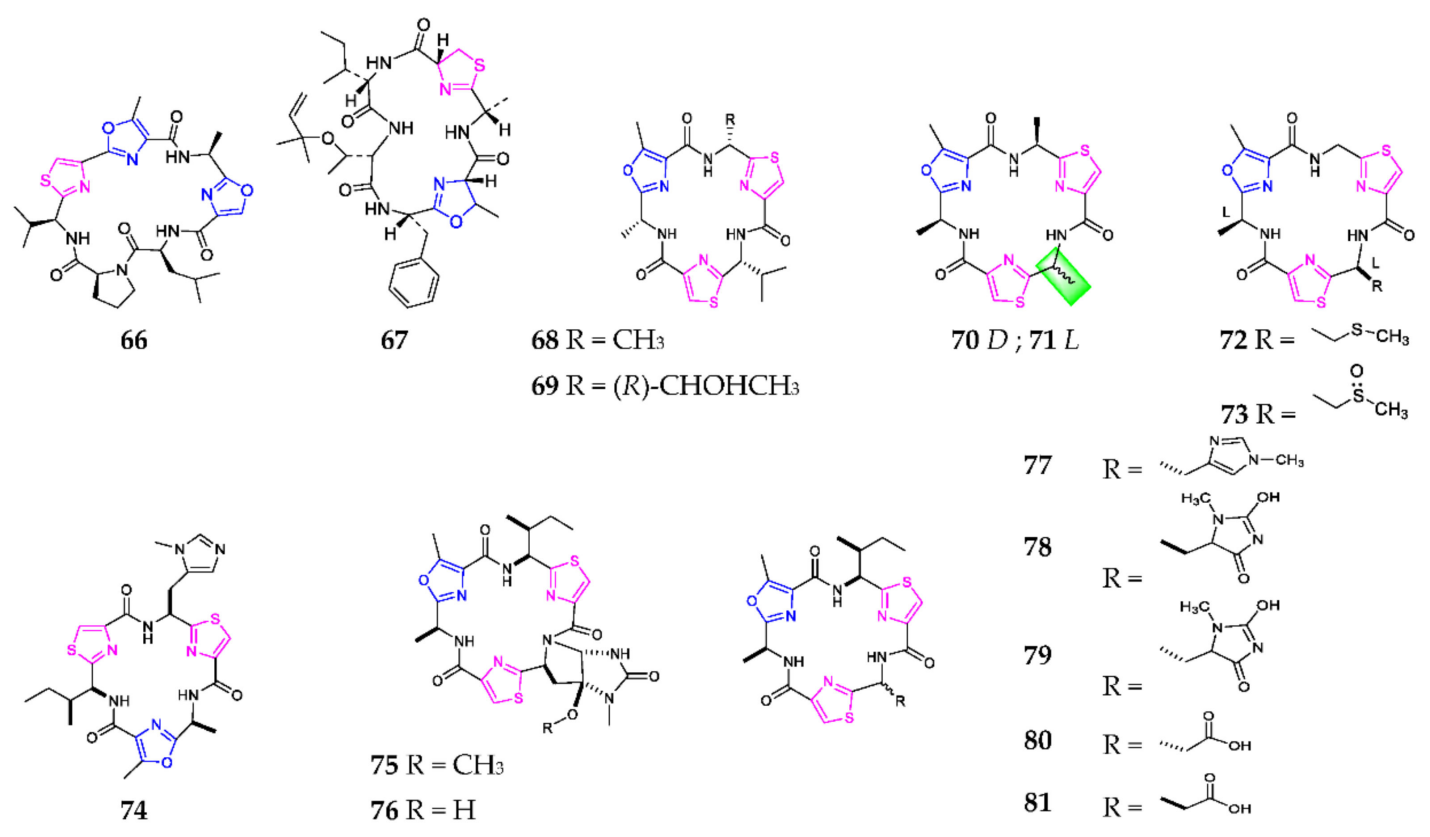
Marine-derived 1,3-oxazole-containing monocyclic hexapeptides (**66**–**81**).

Dolastatins E (**64**) and I (**65**) were obtained from the Japanese sea hare *Dokizbelh auriculariu,* and displayed inconspicuous cytotoxic activity against HeLa S_3_ cells [45,46]. The former substance structurally consisted of one *L*-alanine, one *D*-alanine, one *D*-cysteine, one allo-*D*-isoleucine, one serine and one cysteine [47], and the later was formed by a *N*-Boc-*L*-valine and *N*-Boc-*L*-serine [48]. One cytotoxic 1,3-oxazole-containing hexapeptide, leucamide A (**66**) (Scheme 4), was produced by an Australian sponge *Leucetta microraphis* [49], and its uniquely mixed 4,2-bisheterocycle tandem pair provided antiviral activity through a specific interaction with DNA/RNA or other targets [50]. Furthermore, the construction of the 4,2-bisheterocycle tandem pair was successfully forged by a diethylaminosulfur trifluoride (DAST)-mediated cyclization of *ꞵ*-hydroxy amide in the first total synthesis [51].

One novel cyclic hexapeptide, comoramide A (**67**), produced by the ascidian *Didemnum molle* and collected at Dzaoudzi Mayotte, was found to exhibit mild cytotoxicity against tumor cells A549, HT29 and MEL-28 [52]. Bioassay-guided fractionation of the marine cyanobacterium *Oscillatoria* sp., collected off Buenaventura Bay, led to the isolation of two antimalarial agents, venturamides A (**68**) and B (**69**), which were chemically synthesized using an efficient one-pot method with respective yields of 32.7% and 30.7% [53]. Tenuecyclamides A–D (**70**–**73**) were purified from the cyanobacterium *N. spongiaeforme* var. *tenue*, of which **70**, **72** and **73** had an inhibitory effect on the sea urchin embryos’ division with an ED_100_ of 10.8, 9.0 and 19.1 µM, respectively [54]. The stereochemistry of **70** and **71** was established through the solid-phase assembly of heterocyclic amino acids in 2004 [55]. Microcyclamides are one class of cyclic hexapeptides formed by the ribosomal pathway [56]. Chemical study of the cyanobacterium *Microcystis aeruginosa* afforded eight cytotoxic microcyclamide derivatives (**74**–**81**) [57,58].

#### 2.2.3. Heptapeptides

**Scheme 5:**
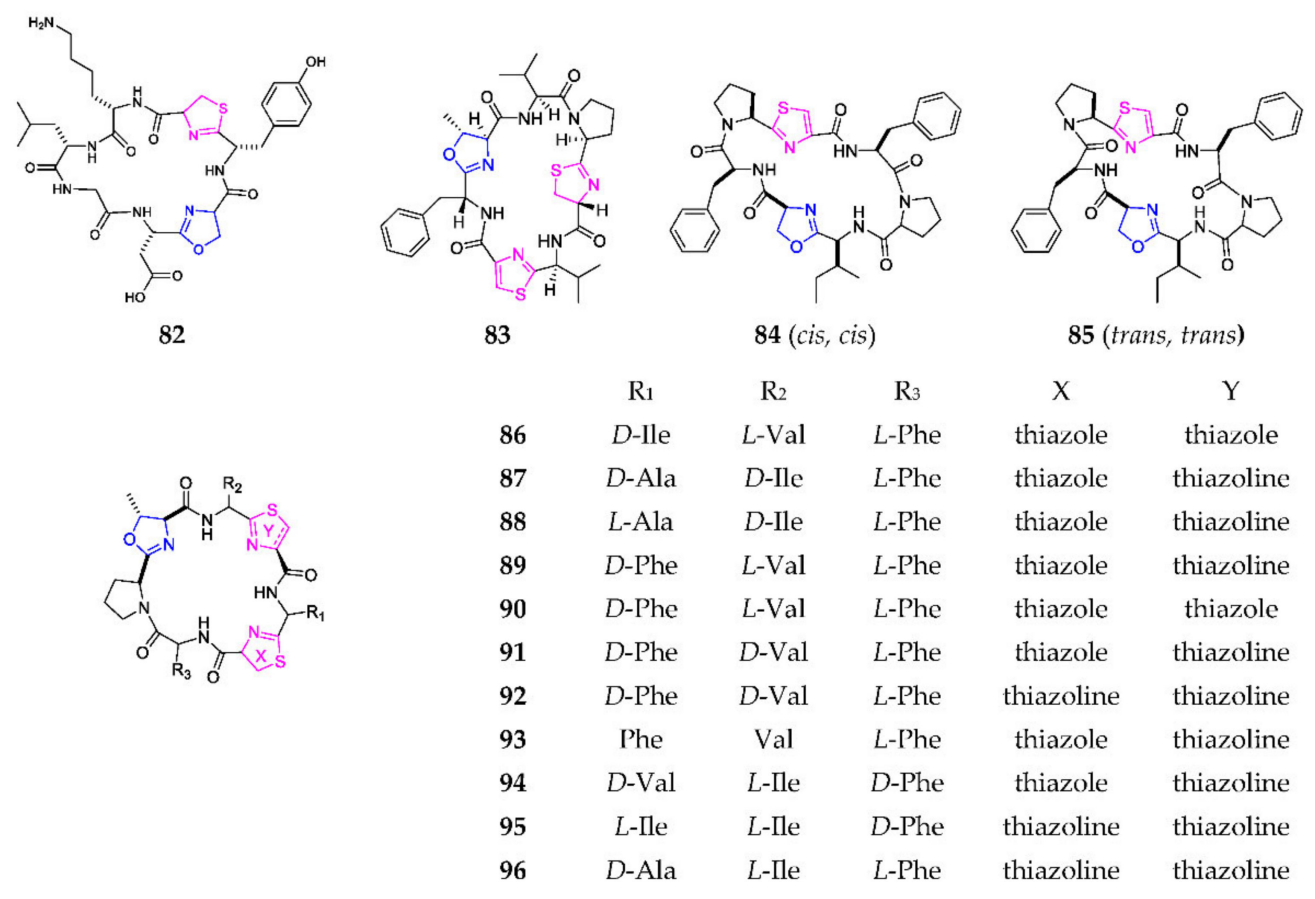
Marine-derived 1,3-oxazole-containing monocyclic heptapeptides (**82**–**96**).

Using the heat shock method, one novel 1,3-oxazole-containing heptapeptide, cyanothecamide C (**82**) (Scheme 5), was produced by the marine cyanobacteria *Cyanothece* sp. PCC 7425 at 37° for 24 h [59]. Cyclodidemnamide (**83**) was derived from the marine ascidian *Didemnum molle,* collected on the Philippine Islands, and exhibited weak cytotoxicity towards human colon tumor cells [60]. Two isomers, (*cis,* *cis*)-ceratospongamide (**84**) and (*trans,* *trans*)-ceratospongamide (**85**), were obtained from the Indonesian red algae *Ceratodictyon spongiosum* and symbiotic sponge *Sigmadocia symbiotica*, of which the latter possessed a potent anti-inflammatory effect with an ED_50_ value of 32 nM [61]. Chemical investigation of the ascidian *Lissoclinum patella* resulted in the sequential isolation of 11 cytotoxic heptapeptides, including lissoclinamides (**86**–**95**) and ulicyclamide (**96**) [62,63,64,65,66,67]. SAR analysis indicated that cytotoxic effects of **89**, **90** and **92** were obviously reduced if the thiazoline ring was replaced by oxazoline, and that of **92** was significantly increased by replacing oxazoline in the macrocycle with thiazoline [68].

#### 2.2.4. Octapeptides

**Scheme 6:**
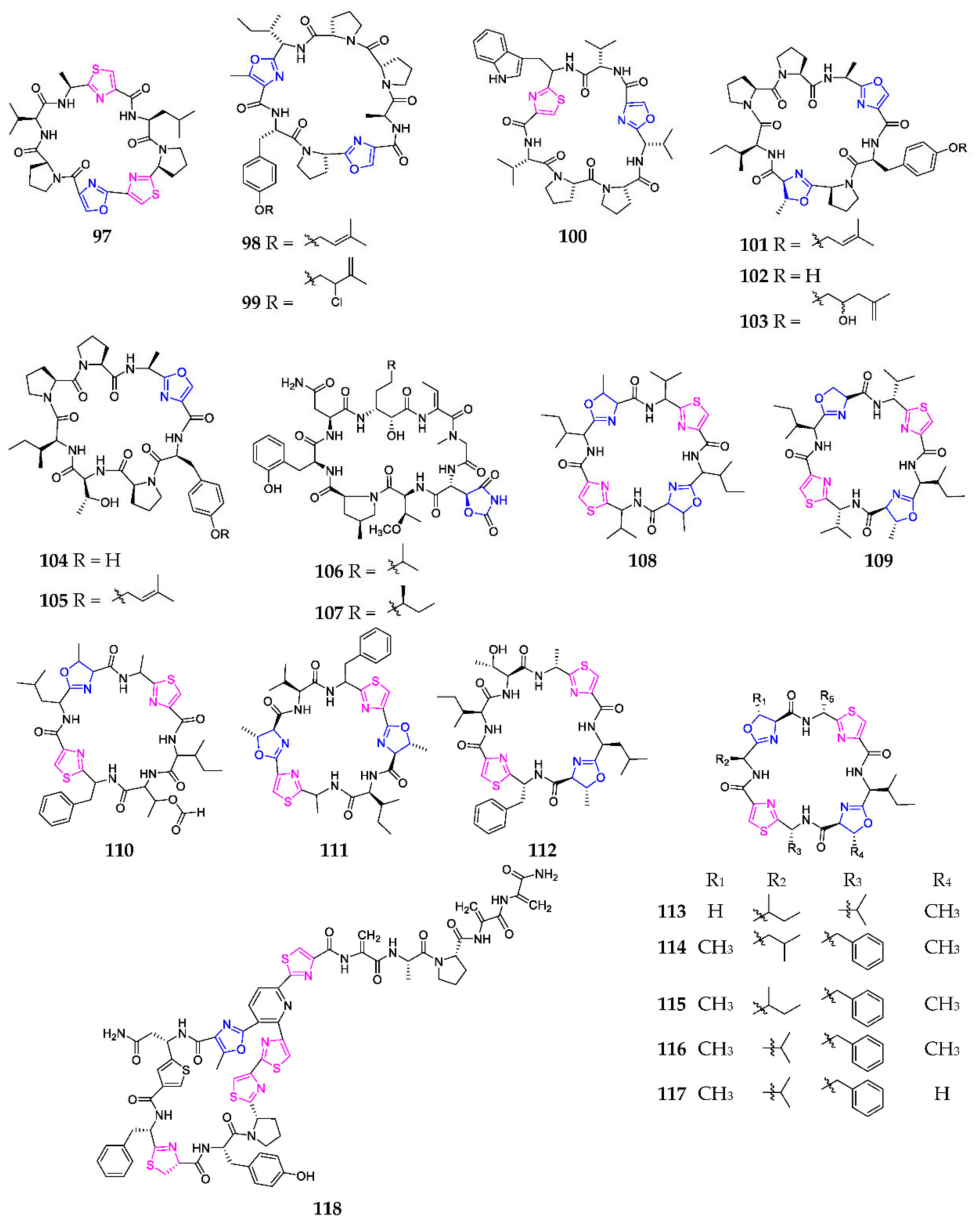
Marine-derived 1,3-oxazole-containing monocyclic octapeptides (**97**–**118**).

Sanguinamide B (**97**) (Scheme 6) was a modified macrocyclic octapeptide containing five *L*-amino acids, two thiazoles and one oxazole from the nudibranch *Hexabranchus sanguineus* [69]. The first total synthesis of **97** was achieved by Singh and coworkers in 2012 and determined its configuration as *trans* [70]. Myriastramides A–C (**98**–**100**) were new modified cyclic peptides purified from the Philippines marine sponge *Myriastra clavosa* [71]. Haliclonamides A–E (**101**–**105**), produced by the marine sponge *Haliclona* sp., displayed an antifouling effect on the blue mussel *Mytilus edulis galloprovincialis* [72,73]. Chemical analysis of the marine sponge *T. swinhoei,* collected on an isolated reef of the Solomon Islands, yielded two anti-inflammatory octapeptides, perthamides J (**106**) and K (**107**) [74]. Ascidiacyclamide (**108**), formed by the cyclization of [Ile-Oxz-*D*-Val-Tz-]_2_, was purified from an unidentified species of ascidian grown on Rodda Reef (Australia) and contains four five-membered heterocycles, where the number and position of oxazoline residues were shown to affect its cytotoxicity [75]. Prepatellamide A (**109**), prepatellamide B formate (**110**) and patellamides A–G (**111**–**117**) were successively obtained from the ascidian *Lissoclinum patella*, of which compounds **109**, **113**, **114** and **111** exhibited modest cytotoxicity against P388 murine leukemia cell lines [76,77,78,79,80,81,82,83,84]. Gene knockout studies indicated that the *Pat* cluster (*pat* A-G) was responsible for patellamide biosynthesis [85]. One anti-MRSA thiazolyl peptide, kocurin (**118**), produced by the marine-derived bacterium *Kocuria palustris,* collected in Florida Keys (Florida, FL, USA), structurally consists of *L*-Tyr, *L*-Pro, *L*-Ala, *L*-Asp, *L*-Phe, *L*-Cya, *L*-Pro (×2), *L*-Ala and *L*-Cys [86]. Biosynthetically, the formation of oxazole and thiazoline rings of **118** were catalyzed by KocE, and its serine residues were dehydrated by KocB and KocC, followed by the Aza–Diels–Alder reaction catalyzed by KocD, resulting in a generation of a 29-membered cycle [87].

#### 2.2.5. Other Monocyclic Peptides

**Scheme 7:**
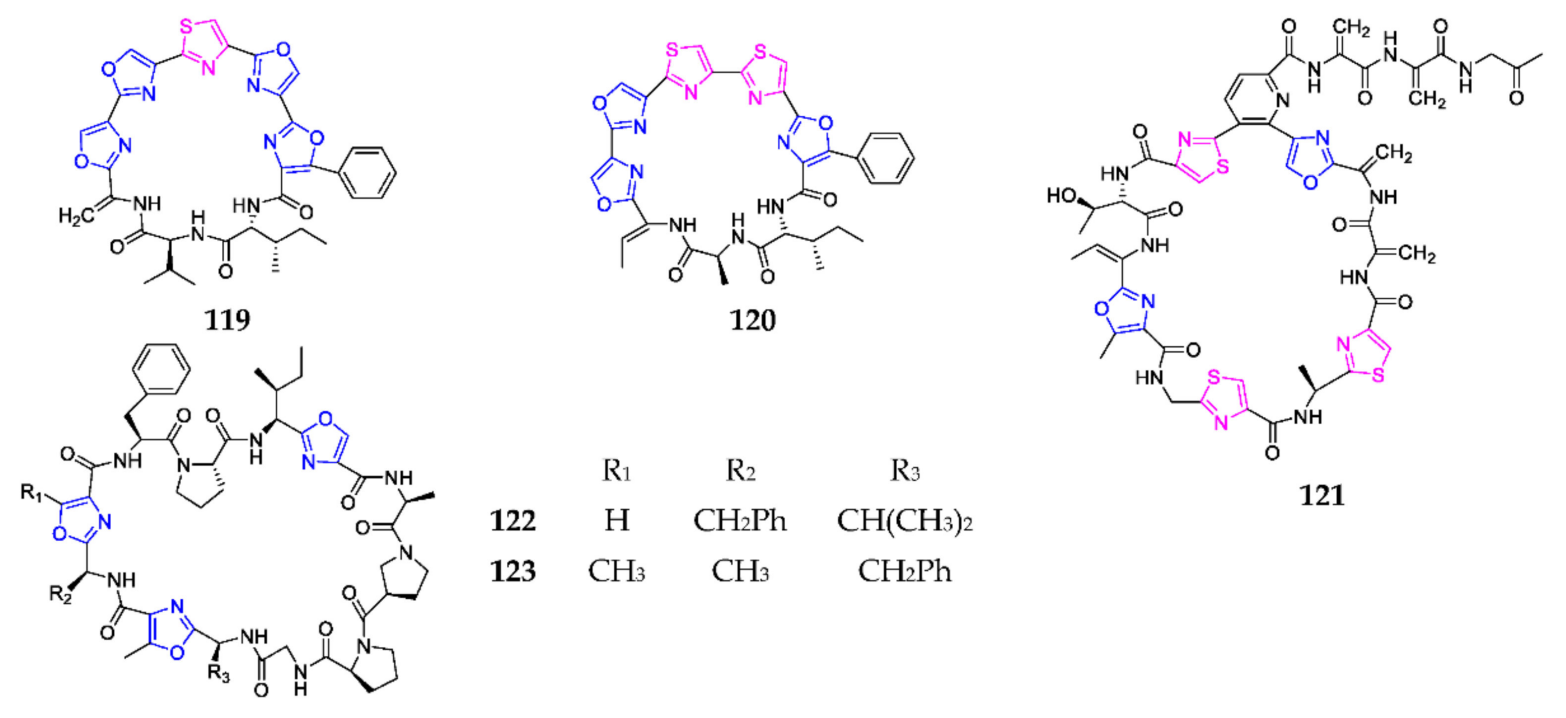
Marine-derived 1,3-oxazole-containing other monocyclic peptides (**119**–**123**).

In addition to mechercharmycin B (**28**), one monocyclic nonapeptide, mechercharmycin A (**119**) (Scheme 7), was purified from the marine strain *Thermoactinomyce*s sp. YM3-251 and shown to have excellent cytotoxicity toward A549 and Jurkat cells, with IC_50_ values of 40 and 46 nM, respectively [29]. Urukthapelstatin A (**120**) was detected in the fermentation broth of the marine strain *Mechercharimyces asporophorigenens* YM11-542, from Urukthapel Island (Palau), and displayed a broad spectrum of potential cytotoxicities [88]. By the macrocyclization of one macrothiolactone, and the azole formatted by the Aza-Wittig ring contraction, chemical synthesis of compound **120** was first achieved by Schwenk and coworkers [89]. Interestingly, the SAR study showed that the phenyl ring attached to the eastern oxazole, and the rigid lipophilic tripeptide section definitely affected its cytotoxicity [90]. One new thiopeptide TP-1161 (**121**) was isolated from one marine *Nocardiopsis* sp. and its biosynthetic gene cluster (BGC) was confirmed by targeted gene inactivation [91]. This substance exhibited no inhibitory effect on Gram-negative bacteria, but excellent antimicrobial activity against a panel of Gram-positive clinical isolates [92]. Wewakazole (**122**) and wewakazole B (**123**) were novel cyclic dodecapeptides produced by cyanobacteria *Lyngbya majuscule* from Papua New Guinea, and *Moorea producens* collected in the red sea, respectively [93]. Bioassay results suggested that compound **122** was active against non-small cell lung cancer H-460 cells (IC_50_ 10.1 μM) and that **123** had a stronger cytotoxicity against H460 cells (IC_50_ 1.0 μM) and MCF7 breast cancer cells (IC_50_ 0.58 μM). The first total synthesis of **123** was achieved through the careful choice of amino acid-protecting groups and the construction of three different substituted oxazoles [94].

### 2.3. Bicyclic Peptides

**Scheme 8:**
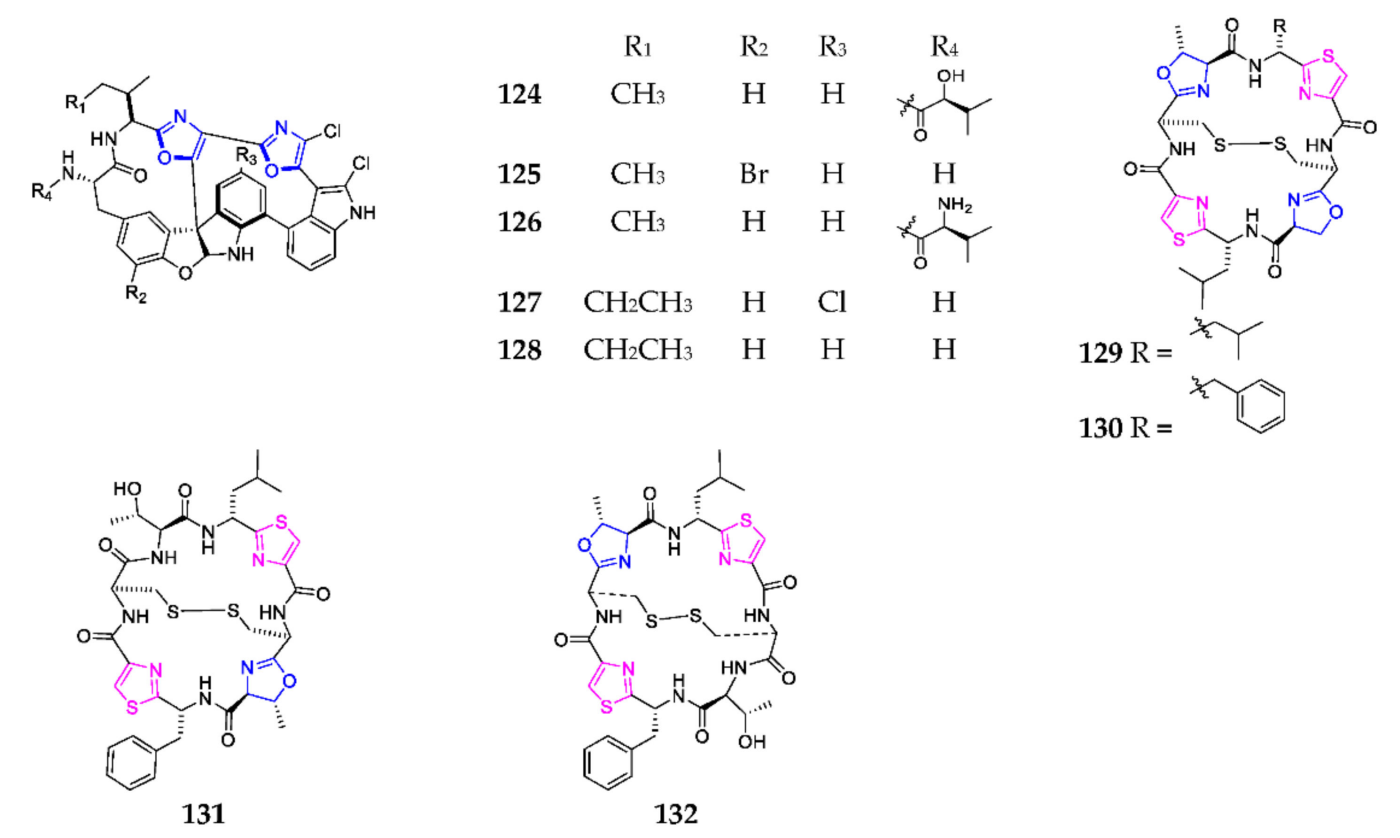
Marine-derived 1,3-oxazole-containing bicyclic peptides (**124**–**132**).

Chemical analysis of the ascidian *Diazona angulate* led to discovery of five new peptides with two 12-membered macrocycles connected by a quaternary carbon stereo center, substituted by triaryl groups in furan indole nuclei, diazonamides A-E (**124**–**128**) (Scheme 8). In particular, compound **124** displayed an extraordinary antitumor effect on a HCT-116 human colon carcinoma and B-16 murine melanoma cancer cell lines, with IC_50_ values less than 15 ng/mL [95,96], and was chemically synthesized by using the Witkop photocyclization reaction for the first time [97]. *Lissoclinum patella* is the prolific producer of the new sulfur-containing bicyclic peptides ulithiacyclamide (**129**), ulithiacyclamide B (**130**), ulithiacyclamide F (**131**) and ulithiacyclamide G (**132**) [67,76,98]. Biological evaluation showed that compounds **129** and **130** excellently inhibited the growth of the KB cell line with an IC_50_ of 35 and 17 ng/mL, respectively.

### 2.4. Depsipeptides

**Scheme 9:**
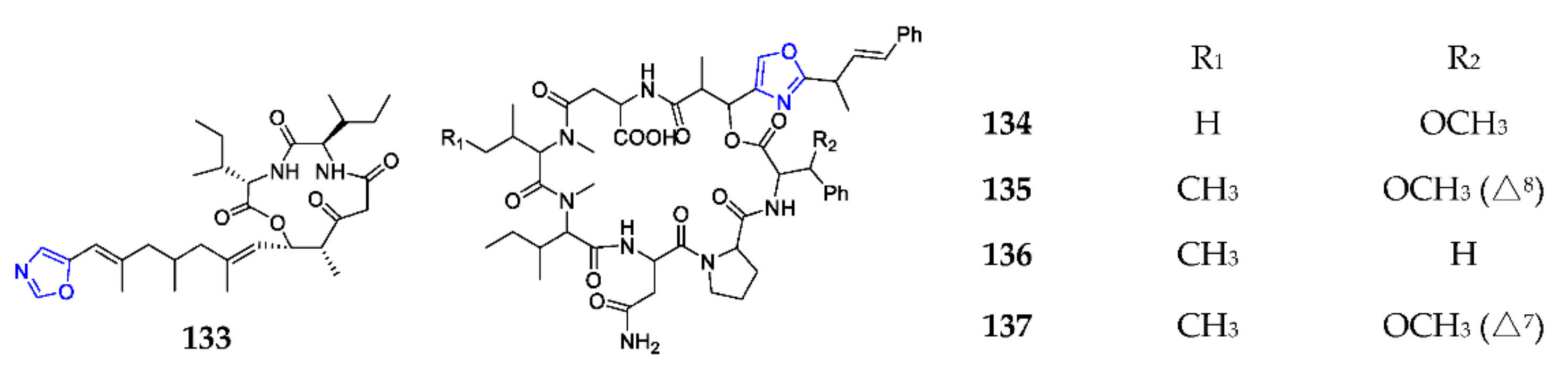
Marine-derived 1,3-oxazole-containing depsipeptides (**133**–**137**).

Taumycin A (**133**) was obtained from the Madagascan sponge *Fascaplysinopsis* sp. and found to inhibit the growth of the human UT-7 leukemic cell line at 1 µM [99]. Total synthesis study indicated that macrocyclization initiated by the thermal rearrangement of *β*-keto *tert*-butyl ester yielded 21% of **133** (Scheme 9) and determined the configuration of C-9 as *S* [100]. Four cyclic depsipeptides, discokiolides A–D (**134**–**137**), were characterized from the marine sponge *Discodermia kiiensis* and exhibited a broad spectrum of cytotoxic activities against P388, P388/ADM, B16-BL6, Lewis, Lu-99, HT-29 and CCD-19Lu with IC_50_ values ranged from 0.5 to 7.2 μg/mL [101].

## 3. Macrolides

### 3.1. Endocyclic Oxazoles

#### 3.1.1. Mono-Oxazole Macrolides

**Scheme 10:**
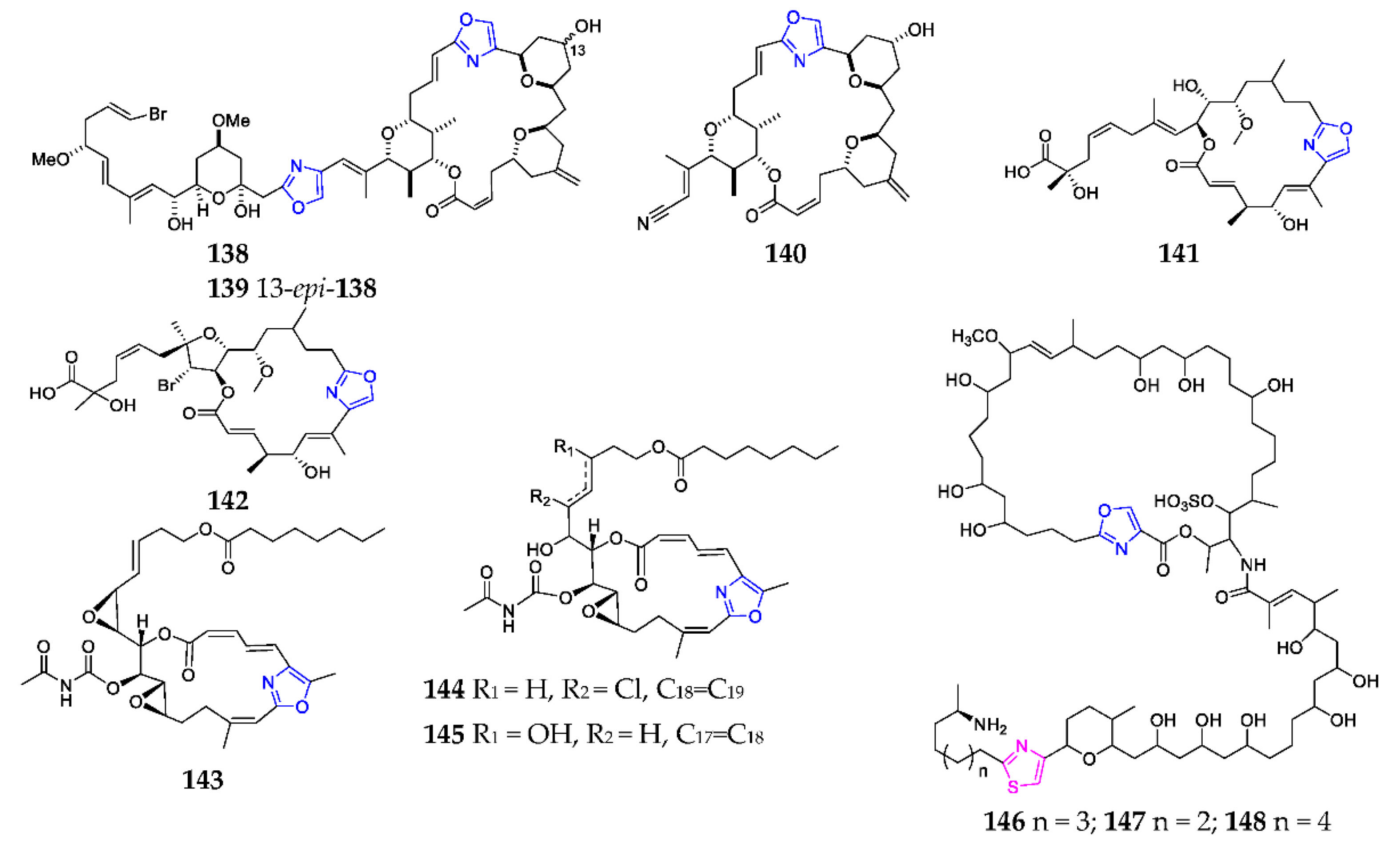
Marine-derived endocyclic mono-oxazole macrolides (**138**–**148**).

Until 2020, marine sponges were the only sources of macrolides containing a mono-oxazole motif. Two cytotoxic isomers, phorboxazoles A (**138**) (Scheme 10) and B (**139**), as well as the precursor (**140**) were detected in the Indian Ocean marine sponge *Phorbas* sp. [102,103]. Leiodolides A (**141**) and B (**142**) were the first members of a new class of mixed polyketide-nonribosomal peptide synthetase from the deep-water marine sponge *Leiodermatium*. They structurally possess a 19-membered ring and several unique functional groups, including a bromine substituent and an α-hydroxy-α-methyl carboxylic acid side-chain terminus. These substances had obvious cytotoxic effects against human colon cancer HCT-116 with IC_50_ values of 2.5 and 5.6 μM, respectively [104]. Chemical investigation of the Madagascan sponge *Fascaplysinopsis* sp. afforded three macrolides with bis-epoxide motif salarins **143**–**145**, which showed pronounced inhibitory on human leukemia cell lines UT-7 [105,106]. Theonezolides A–C (**146**–**148**) from the Okinawan *Theonella* sp. were novel oxazole-containing macrolides that compose of two main fatty acid chains, including a 37-membered macrolide ring with long side chains connected by amide bonds. They exhibited cytotoxicity on a murine lymphoma L1210 and human epidermoid carcinoma KB cells and induced a platelet morphology change and aggregation in rabbits [107,108,109].

#### 3.1.2. Dioxazole Macrolides

**Scheme 11:**
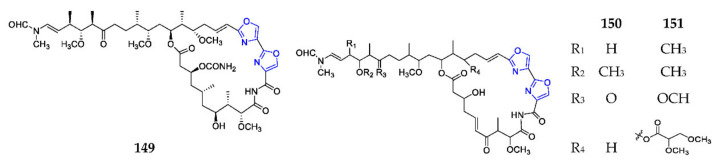
Marine-derived endocyclic dioxazole macrolides (**149**–**151**).

Three 25-membered macrolides consisted of two consecutive oxazole rings, kabiramide I (**149**) (Scheme 11), halishigamide B (**150**) and mycalolide D (**151**), were respectively isolated from the Thai sponge *Pachastrissa nux*, the Okinawan *Halichondria* sp., and the stony coral *Tubastrea faulkneri*, of which compound **150** had a weak cytotoxicity against L1210 and a modest antifungal effect on *T. mentagrophytes*, while **151** showed a modest general cytotoxicity with average LC_50_ values of 0.6 µM against the NCI’s 60-human tumor cell line [110,111,112].

#### 3.1.3. Trioxazole Macrolides

**Scheme 12:**
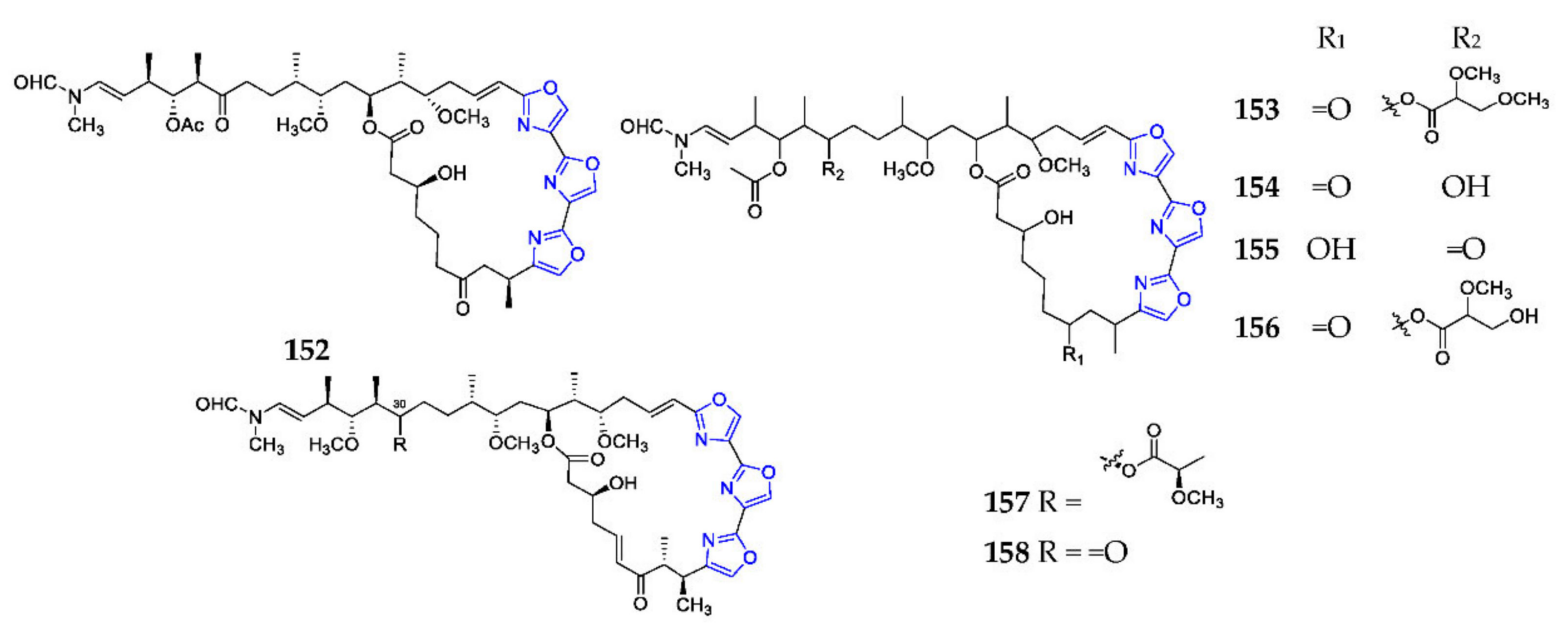
Marine-derived endocyclic trioxazole macrolides (**152**–**158**).

Trioxazole macrolides with extended aliphatic tails are an unprecedented class of natural products and have not been found in terrestrial organisms before [113]. Ulapualides A–E (**152**–**156**) (Scheme 12) were extracted from the nudibranch *Hexabranchus sanguineus,* and compounds **152** and **153** displayed an inhibitory effect on the L1210 leukemia cell with IC_50_ values ranged from 0.01 to 0.03 µg/mL [114]. Miuramides A (**157**) and B (**158**) of the *Mycale* sp. sponge collected from Miura Peninsula were shown to possess potent cytotoxicities against 3Y1 cells in the nM range [115].

**Scheme 13:**
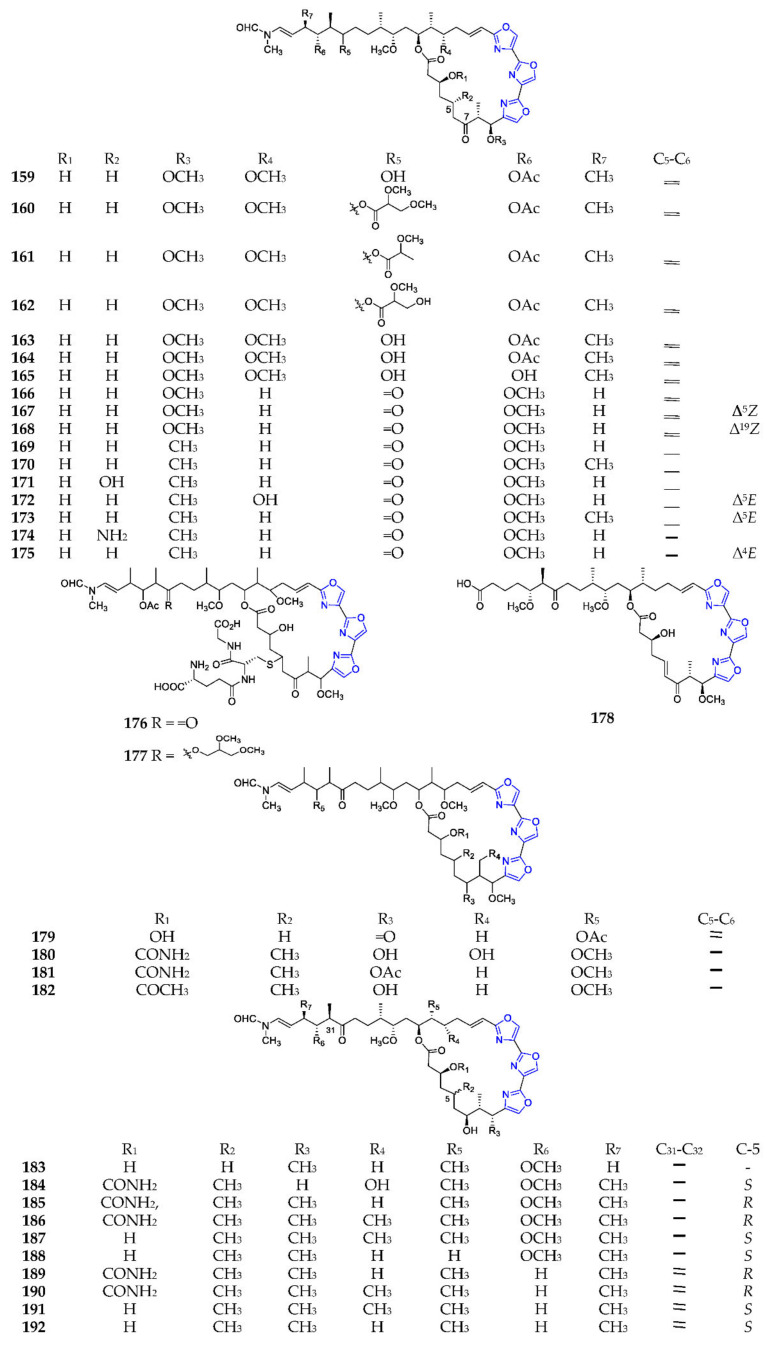
Marine-derived endocyclic trioxazole macrolides (**159**–**192**) (Cont.).

Mycalolides A–C (**159**–**161**) (Scheme 13) and their derivatives (**162**–**165**, **176**, **177**, **179**) were obtained from a marine sponge of the genus *Mycale,* except mycalolide E (**166**) from the coral *Tubastrea faulkneri*, of which **176** and **177** possessed one sulfur atom connected to the glutathionyl group (i-Glu-Cys-Gly) at C-5 [116]. Bioassay revealed that these substances exhibited excellent cytotoxicities [117]. Phytochemical analysis of sponges *Halichondria* sp., *Jaspis* sp., *Chondrosia corticata* and nudibranch *Hexabranchus sanguineus* resulted in the discovery of thirteen halichondramides (**166**–**181**, **183**, **184**), which had significant antifungal and cytotoxic effects [111,118]. Kabiramides (**180**–**182**, **185**–**192**) were discovered in the *Hexabranchus* egg masses and the sponge *Pachastrissa nux,* and displayed extraordinary cytotoxicities, and antiplasmodial and antifungal activities [119].

### 3.2. Exocyclic Oxazoles

**Scheme 14:**
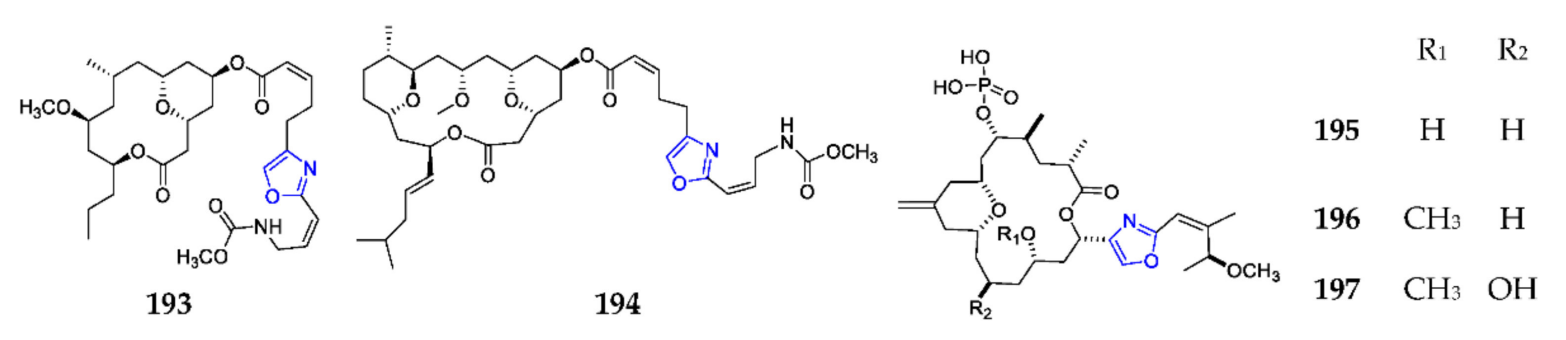
Marine-derived exocyclic oxazoles (**193**–**197**).

Neopeltolide (**193**) (Scheme 14) was one of the new exocyclic oxazole-containing macrolides from a deep-water sponge of the family *Neopeltidae*, and was found to have potential antifungal effects against *C.* *albicans* and inhibitory activities against A-549, NCI-ADR-RES and P388 cell lines with IC_50_ values of 1.2, 5.1 and 0.56 nM, respectively [120]. SAR analysis revealed that the substitution of the side chain and the stereochemistry of the macrolide carbon, especially C-11 and C-13, is vital for the overall biological property [121]. One doubly O-bridged 18-membered macrolide, leucascandrolide A (**194**), was detected in a calcareous sponge of a new genus *Leucuscundra caveoluta* from the Coral Sea [122]. Pharmacological study indicated that this metabolite was a novel inhibitor of cytochrome *bc*_1_ [123]. Enigmazoles **195**–**197,** from the sponge *Cinachyrella enigmatica,* were a new structural family of marine phosphomacrolides with an 18-membered macrocyclic ring, which consists of an embedded 2,6-*cis*-substituted tetrahydropyran ring, a 2,4-disubstituted oxazole ring in the side chain, and a phosphate group. These substances can inhibit cells with either a wild-type or mutant c-Kit. However, their actual cellular targets are still unknown [124]. The first chemical synthesis of **195** and **196** required a tandem olefin cross-metathesis/intramolecular oxa-Michael addition reaction [125].

## 4. Polyketides

### 4.1. Mono-Oxazole Polyketides

**Scheme 15:**
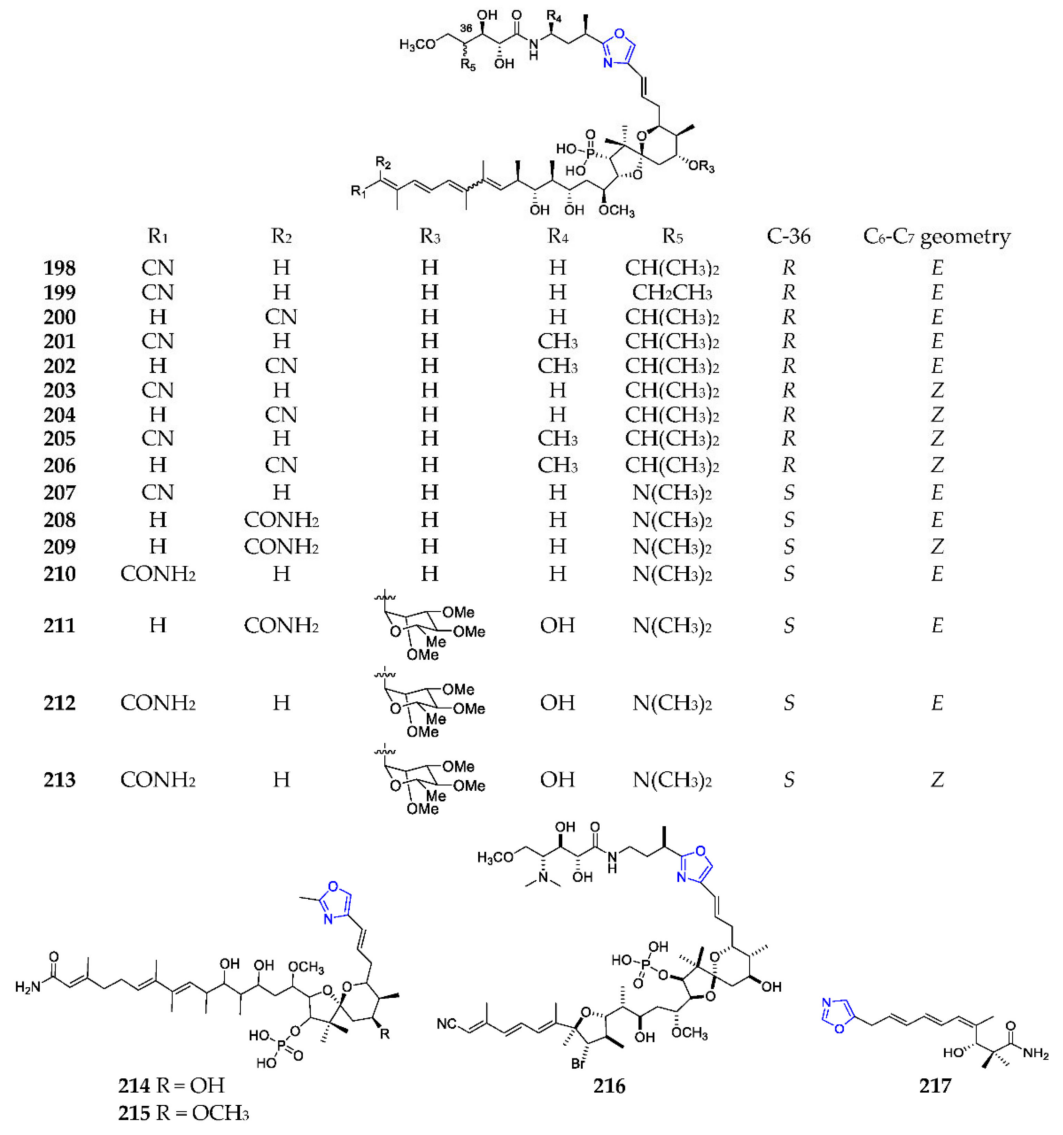
Marine-derived mono-oxazole polyketides (**198**–**217**).

Calyculins (**198**–**216**) (Scheme 15) were novel polyketides containing a mono-oxazole and a phosphate group from the marine sponges *Discodermia calyx*, *Lamellomorpha strongylat*, *Luffariella geometrica*, *Myriastra clavosa* and *Theonella swinhoei*. These natural products exhibited extensive biological properties including cytotoxicity, antifungal activity and an inhibitory effect on protein phosphatases [126,127,128,129,130,131]. By OSMAC (one strain many compounds) strategy, inthomycin B (**217**) was produced by the marine sediment-derived *Streptomyces* YB104 and was found to have anti-oomycete, cytotoxic and herbicidal activities [132]. One concise method to synthesize **217** was developed by Webb and coworkers through the Stille coupling of a stannyl-diene with an oxazole vinyl iodide unit and a Kiyooka ketene acetal/amino acid-derived oxazaborolidine procedure as its cornerstones [133]. And the gene cluster (*itm*) responsible for biosynthesis of **217** was identified as a 95.3 kb trans-AT type I PKS system, of which the gene *Itm*15 is a cyclodehydrase to catalyze the formation of oxazole ring [132].

### 4.2. Dioxazole Polyketides

**Scheme 16:**
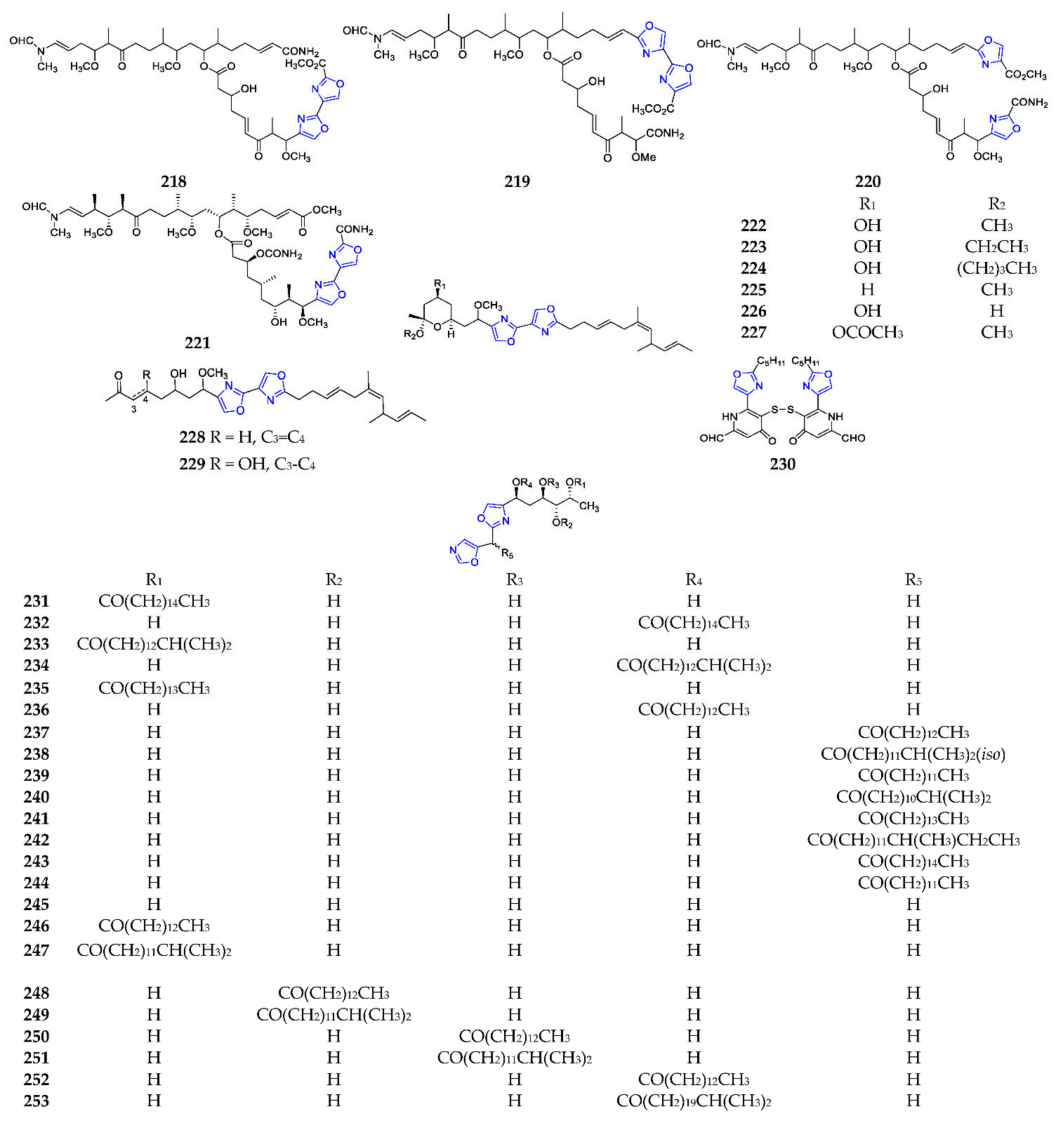
Marine-derived dioxazole polyketides (**218**–**253**).

Four analogs of the trioxazole macrolide **169** linked with a methyl ester and a primary amide group, secohalichondramide (**218**) (Scheme 16), halishigamides C (**219**) and D (**220**), and kabiramide H (**221**), were isolated from marine sponges, including *Chondrosia corticate*, *Halichondria* sp. and *Pachastrissa nux* [110,111,134]. Metabolites **219** and **220** showed weak cytotoxicity against L1210 and KB cell lines and a modest antifungal activity against *T. mentagrophytes* [111]. By extensive chromatographic techniques, eight hennoxazoles (**222**–**229**) were isolated from the sponge *Polyfibrospongia* sp., of which **222** had peripheral analgesic activity equivalent to the positive control of indomethacin, and **228** exhibited the greatest cytotoxicity toward L1210 with an IC_50_ value of 2 µg/mL [135,136]. Antibiotic B-90063 (**230**) was a novel endothelin-converting enzyme (ECE) inhibitor from the marine strain *Blastobacter* sp. SANK 71894, collected off the coast of Ojika Peninsula [137]. Bengazoles **231**–**236** were antifungal agents obtained from the marine sponge *Pachastrissa* sp., collected at Musha Archipelago (Djibouti) [138]. An uncommon oxazole, bengazole A (**237**) from the sponge *Jaspis* sp., displayed remarkable ergosterol-dependent antifungal activity against *C. albicans*, which is equivalent to amphotericin B [139].

## 5. Benzoxazoles

**Scheme 17:**
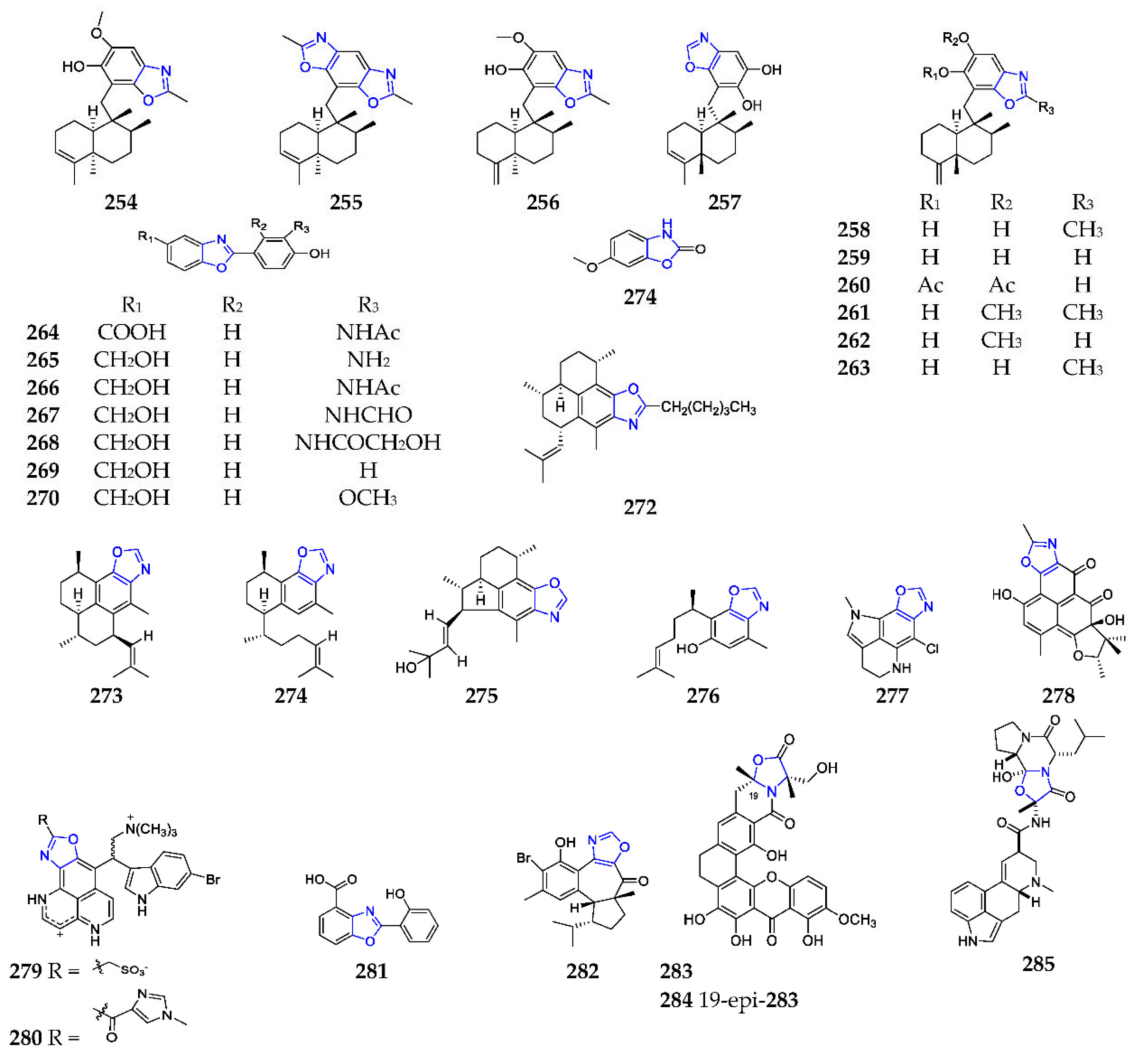
Marine-derived benzoxazoles (**254**–**285**).

Benzoxazole-containing compounds are one important class of natural products with a potential application in pharmaceutical and agrochemical fields [140,141]. Ten sesquiterpenoid benzoxazoles (**254**–**263**) have been successively purified from marine sponges since 1995, including the Okinawan sponge of the family *Spongiidae*, the *Smenospongia* sp., the *Hyrtios* sp., and the *Dactylospongia* sp. [142,143,144,145]. A novel approach to totally synthesize these substances was developed through the cyclic closure of the *N*-(2-hydroxyphenyl)-formamide or -acetamide groups to obtain the desired dihydroxybenzoxazole substructure [146]. Chemical study of a halophilic strain *Nocardiopsis lucentensis* DSM 44048 yielded seven new benzoxazoles, nocarbenzoxazoles A–G (**264**–**270**) (Scheme 17), in which **270** had selective activity against HepG2 and HeLa cell lines with a IC_50_ of 3 and 1 μM, respectively [147]. Recently, a simple route to the synthesis of **269** and **270** was achieved by microwave-assisted construction of a benzoxazole skeleton [148]. One 6-methoxy-2(3*H*)-benzoxazolinone, coixol (**271**), was detected in the DCM-MeOH extract of the marine sponge *Oceanapia* sp., collected off Mandapam coast (India), and had extensive bioactivities, including an inhibitory effect on brine shrimp, anti-inflammation and anti-diabetes [149]. Bioassay-guided fractionation of an extract from the sea plum *Pseudopterogorgia elisabethae* led to the isolation of homopseudopteroxazole (**272**), pseudopteroxazole (**273**), *seco*-pseudopteroxazole (**274**) and ileabethoxazole (**275**) [150,151]. Oxazocurcuphenol (**276**) was isolated and characterized from the coral *P. rigida,* collected at Lighthouse Point on the Eleuthera island (Nassau, Bahamas) [152]. Biological tests suggested that **272** and **273**–**275** strongly inhibited the growth of *Mycobacterium tuberculosis* H_37_R0v at 12.5 µg/mL. Compounds **272** and **273** were chemically synthesized by a rapid one-pot method [153], and the total synthesis of **275** was accomplished using a new enantioselective approach [154].

To the best of our knowledge, citharoxazole (**277**) was the first chlorinated oxazole-containing pyrrolo[4,3,2-de]quinoline from the Mediterranean deep-sea sponge *Latrunculia* (*Biannulata*) *citharistae* [155]. Culture of the marine fungus *Penicillium* sp. from a marine sediment resulted in the production of herqueioxazole (278), which was the first oxazole-containing phenalenone [156]. The marine sponge *Suberites* sp. was the unique source to produce nakijinamines C (**279**) and E (**280**) with a 1*H*-oxazolo[40,50:4,5]benzo[1,2,3-de] [1,6]naphthyridine ring system [157]. One new benzoxazole caboxamycin (**281**), from the deep-sea strain *Streptomyces* sp. NTK 937, was found to strongly inhibited phosphodiesterases [158]. Lately, one facile method for synthesis of this antibiotic has been developed via debenzylation and demethylation, under eco-friendly and simple reaction conditions [159], and one regulatory and nine structural genes involved in its biosynthesis have been confirmed through genetic manipulation [160]. Hamigeran M (**282**) was the first non-benzo-fused oxazole-containing terpenoid from the New Zealand marine sponge *Hamigera tarangaensis*, and exhibited the high efficacy against the HL-60 promyelocytic leukemia cell line with a mean IC_50_ value of 6.9 ± 0.4 μM [161]. Two novel anti-MRSA xanthones, citreamicins θA (**283**) and θB (**284**), were separated from one marine-derived strain of *Streptomyces caelestis* [162]. Ergosinine (**285**) was one ergot alkaloid possessing an oxazole ring from the sea slug *Pleurobranchus forskalii,* collected off Ishigaki Island (Ishigaki, Japan) and found to have an anti-adrenergic effect [163].

## 6. Conclusions

In recent decades, a tremendous number of 1,3-oxazole-containing alkaloids have been isolated and characterized from marine organisms, including marine invertebrates, nematodes, insects, vertebrates cyanobacteria, bacteria, and fungi. These substances possess unique chemical structures and exhibit a wide variety of biological properties. Many important marine-derived 1,3-oxazoles have great potential for the development of leading compounds in the search for new drugs and medicines. For example, mechercharmycin A (**119**) is a promising antitumor remedy, and (19Z)-halichondramide (**168**), neohalichondramide (**175**), and calyculin A–D (**190**, **200**–**202**) have the potential to treat human leukemia cell line. Besides, plenty of these substances are potential antimicrobial agents, such as kocurin (**118**), TP-1161 (**121**) homopseudopteroxazole (**272**), and so on. However, (1) how to reduce cytotoxicity, (2) how to improve drug stability, and (3) how to make the drug work quickly and effectively in vivo still needs to be studied in further depth.

In recent years, however, the number of new 1,3-oxazole derivatives from marine organisms has greatly decreased, since almost all accessible macroorganisms have been collected and chemically analyzed. Simultaneously, marine microorganisms are shown to be one prolific and unexploited source of bioactive natural products, owing to their species richness and abundant secondary metabolite BGCs, especially symbiotic microbes of marine sponges, seaweeds, mangroves and tunicates [164,165,166,167]. In order to discover novel marine microbe-derived 1,3-oxazoles for new drug discovery, more efforts should be made to conduct strain isolation and chemical study using a combination of classical methods (e.g., cultivation and fermentation, bioassay-guided fractionation, structure elucidation) and advanced analytical techniques (e.g., metabolomics, higher-field NMR instruments, probe technology), genome mining and engineering and microbial cultivating systems (e.g., OSMAC approach) [168].

## Data Availability

Detail information for all 1,3-oxazole-containing alkaloids described in this work is available at Table S1.

## Supplementary Materials

The following are available online at https://www.mdpi.com/article/10.3390/ph14121274/s1. Detailed information for 1,3-oxazole-containing alkaloids (**1**–**285**) from marine organisms is available in Table S1.

## Abbreviations

ADP	Adenosine diphosphate
Ala	Alanine
Anti-MRSA	Anti-methicillin-resistant *Staphylococcus aureus*
Asp	Aspartate
ATP	Adenosine triphospha
BGC	Biosynthetic gene cluster
Cys	Cysteine
DCM	Dichloromethane
DPPH	1,1-Diphenyl-2-picrylhydrazyl
ECE	Endothelin-converting enzyme
ED_50_	Median effective dose
FMN	Flavin mononucleotide
FMNH_2_	Flavin mononucleotide reduced
Glu	Glutamic acid
Gly	Glycine
IC_50_	Half maximal inhibitory concentration
Ile	Isoleucine
LC_50_	Median lethal concentration
MIC	Minimum inhibitory concentration
NMR	Nuclear magnetic resonance
NCI	National Cancer Institute
OSMAC	One strain many compounds
Phe	Phenylalanine
PKS	Polyketide synthase
Pro	Proline
SAR	Structure-activity relationship
*S. aureu*	*Staphylococcus aureus*
Tyr	Tyrosine
Val	Valine
