# Supplementary material for: An Overview of Bioactive 1,3-Oxazole-Containing Alkaloids from Marine Organisms
Source: Pharmaceuticals (Basel). 2021 Dec 6;14(12):1274. doi: 10.3390/ph14121274 (PMC8706051; doi:10.3390/ph14121274)
Supplement: Supplementary file 1 [file pharmaceuticals-14-01274-s001.zip › pharmaceuticals-1467270-supplementary.pdf]

Supporting Information for

An overview of Bioactive 1,3-oxazole-Containing Alkaloids from Marine Organisms

Jinyun Chen [1], Sunyan Lv [1], Jia Liu [1], Yanlei Yu [2], Hong Wang [1], Huawei Zhang [1,*]

[1] School of Pharmaceutical Sciences, Zhejiang University of Technology, Hangzhou, 310014, China; chenjinyun07@163.com (J.C.); lvsunyan@163.com (S.L.); jliu3092@163.com (J.L.); hongw@zjut.edu.cn (H.W.)

[2] Collaborative Innovation Center of Green Pharmaceutics of Delta Yangzi Region, Zhejiang University of Technology, Hangzhou 310014, China; yanleiyu@zjut.edu.cn

[*] Correspondence: hwzhang@zjut.edu.cn

Contents

Table S1 Detail information for bioactive 1,3-oxazole-containing alkaloids (**1-285**) from marine organisms S2

References S18

Table S1 Detail information for bioactive 1,3-oxazole-containing alkaloids (**1-285**) from marine organisms.

Number	Name	Marine Source	Biological Activity	Reference
1	Almazole A	Red seaweed of the family *Delesseriaceae* from the coasts of Senegal.	--	[1]
2	Almazole B		--	
3	Almazole C		--	[2]
4	Almazole D		Antibacterial against Gram-negative *Serratia marcescens* and *Salmonella typhi* XLD.	[3]
5	Streptochlorin	Strain *Streptomyces* sp. 04DH110 isolated from shallow water sediment taken at -1 m depth of Ayajin Bay (Korea).	Inhibitory activity against human leukemia cells (IC_{50} 1.05 μg/ml) and immortalized hepatocytes derived from normal human liver; antifungal activity.	[4]
6	Martefragin A	Red alga *Martensia fragilis*, collected off the coast of Uozu, Toyama Prefecture (Japan).	Inhibitory activity on NADPH-dependent lipid peroxidation in rat liver microsomes.	[5]
7	Almazolone	Red alga of Senegal *Haraldiophyllum* sp. from Dakar (Senegal).	--	[6]
8	Serratiochelin A	Isolated from a co-culture of *Serratia* sp. and *Shewanella* sp.	Antiproliferative activity on human melanoma cell line and non-malignant lung fibroblasts; antimicrobial effect on *Staphylococcus aureus*.	[7]
9	JBIR 34	A sponge-derived *Streptomyces* sp. Sp080513GE-23, isolated from a marine sponge, *Haliclona* sp. Which collected from Tateyama city, Chiba Prefecture, Japan.	DPPH radical scavenging activity with an IC_{50} value of 1.0 mM.	[8]
10	JBIR 35		DPPH radical scavenging activity with an IC_{50} value of 2.5 mM.	
11	Nigribactin	Marine bacteria *Vibrio nigripulchritudo*, isolated from marine Galathea.	--	[9]
12	Phorbazole A	Marine sponge *Phorbas aff. clathrata* collected off Sodwana Bay (South Africa).	--	[10]
13	Phorbazole B		--	
14	Phorbazole C		--	
15	Phorbazole D		--	
16	9-Chloro-phorbazole D	Nudibranch *Aldisa andersoni*, collected off	*In vitro* growth inhibitory activity against five human cancer cell	[11]

17	*N*1-methyl-phorbazole A	Muttom coast (India).	lines (A549; MCF-7; SKMEL-28; Hs683; U373).	
18<br>19	Ariakemicin A<br>Ariakemicin B; Δ11-isomer	*Rapidithrix* sp. from the muddy land alongside the Ariake Inland Sea in southwest Japan.	Selectively inhibitory effect on the growth of Gram-positive bacteria (*Brevibacterium* sp., *Staphylococcus aureus*, and *Bacillus subtilis*); slight cytotoxicity against A549 human lung cancer cells and BHK baby hamster kidney cells with IC_{50} values of 25 and 15 μg/mL, respectively.	[12]
20	Breitfussin A	Marine hydrozoan *Thuiaria breitfussi*, collected from Bjørnøya (Bear island).	--	[13]
21	Breitfussin B		--	
22	Breitfussin C		Cytotoxicity against seven cancer cell lines (MCF-7, MDA-MB-468, and SK-BR-3; breast adenocarcinoma, HT-29; colon adenocarcinoma, MOLT-4 and MV-4–11; leukemia, and A2058; melanoma) and one nonmalignant cell line (MRC-5; lung fibroblasts); an extraordinarily potential inhibitor for PIM1 and DRAK1 kinases.	[14]
23	Breitfussin D		--	
24	Breitfussin E		---	
25	Breitfussin F		--	
26	Breitfussin G		--	
27	Breitfussin H		--	
28	Mechercharstatin B	Strain *Thermoactinomyces* sp. YM3-251 isolated from sea mud collected at Mecherchar in the Republic of Palau.	--	[15]
29	Siphonazole A	*Herpetosiphon* sp. from a mud sample of the	Selective cytotoxicity to human breast cancer HTB-129 and acute T	[16]

30	Siphonazole A; 3'-Me ether	intertidal region.	cell leukemia TIB-152.	
31	Nazumazole A	Marine sponge *Theonella swinhoei*, collected at Hachijo Island (Japan).	Cytotoxic to P388 cells.	[17]
32	Nazumazole B			
33	Nazumazole C			
34	Nazumazole D		Inhibit chymotrypsin.	[18]
35	Nazumazole E			
36	Nazumazole F			
37	Orbiculamide A	Marine sponge *Theonella* sp., collected off Hachijo Island (Japan).	Cytotoxic to P388 murine leukemia cells.	[19]
38	Keramamide B	Marine sponge *Theonella* sp. collected off Kerama Islands (Japan).	Inhibited the superoxide generation response of the human neutrophils elicited with a chemotactic peptide, *N*-formyl-Met-Leu-Phe (fMLP).	[20]
39	Keramamide C			
40	Keramamide D			
41	Keramamide E		Cytotoxicity against L1210 murine leukemia cells and KB human epidermoid carcinoma cells.	[21]
42	Keramamide M			[22]
43	Keramamide N			
44	Discobahamin A	Marine sponge *Discodermia* sp., isolated from a new species of the Bahamian deep water.	Inhibitor of the growth of *Candida albicans.*	[23]
45	Discobahamin B			
46	Bistratamide A	Ascidian *Lissoclinum bistratum*, collected at Heron Island Reef on the Great Barrier Reef, Australia.	Cytotoxicity toward human cell lines, MRC5CV1 fibroblasts and T24 bladder carcinoma cells.	[24]
47	Bistratamide B			
48	Bistratamide C	Ascidian *Lissoclinum bistratum* in the Philippines.	Depressant effects in the ic. Mouse.	[25]
49	Bistratamide D			
50	Bistratamide E		Moderately cytotoxic in the HCT-116 cell line assay.	[26]
51	Bistratamide F		Moderately cytotoxic in the HCT-116 cell line assay.	
52	Bistratamide G		Moderate activity against the human colon tumor (HCT-116) cell line.	
53	Bistratamide H			
54	Bistratamide I			
55	Bistratamide K		--	[27]
56	Bistratamide L		--	

57<br>58	Bistratamide M<br>Bistratamide N	Ascidian *Lissoclinum bistratum* collected in Raja Ampat (Papua Bar, Indonesia).	Moderate cytotoxicity against four human tumor cell lines with GI_{50} values in the micromolar range.	
59	Westiellamide	A terrestrial blue-green alga *Westiellopsis prolrfia*, collected from a mud sample on the island of Oahu, Hawaii.	Cytotoxic against KB and LoVo cell lines.	[28]
60<br>61<br>62	Dendroamide A<br>Dendroamide B<br>Dendroamide C	A terrestrial blue-green alga (cyanobacterium) *Stigonema dendroideum* Fremy, isolated from a rock sample collected at Wainapanapa, Maui.	Multidrug-resistance reversing activity.<br>--<br>--	[29]
63	Didmolamide A	Ascidian *Didemnum molle*, collected in Madagascar.	Mildly cytotoxic against tumor cell lines (A549, HT29, and MEL28).	[30]
64<br>65	Dolastatin E<br>Dolastatin I	Sea hare *Dolabella auricularia* in Japanese.	Cytotoxic activity against HeLa S_3 cells.<br>Cytotoxicity against HeLa S_3 cells.	[31]<br>[32]
66	Leucamide A	Marine sponge *Leucetta microraphis* collected in Australian.	Moderately cytotoxic toward several tumor cell lines.	[33]
67	Comoramide A	Ascidian *Didemnum molle*, collected at Dzaoudzi Mayotte.	Mild eytotoxicity against several tumor cells (A549, HT29 and MEL-28).	[34]
68<br>69	Venturamide A<br>Venturamide B	Cyanobacterium *Oscillatoria* sp., collected from Buenaventura Bay.	Strong *in vitro* activity against *Plasmodium falciparum* (8.2 μM); mild cytotoxicity to mammalian Vero cells (86 μM).<br>Strong antimalarial activity against *Plasmodium falciparum* (5.6 μM); mild cytotoxicity to mammalian Vero cells (56 μM).	[35]
70<br>71<br>72<br>73	Tenuecyclamide A<br>Tenuecyclamide B<br>Tenuecyclamide C<br>Tenuecyclamide D	Cyanobacterium *Nostoc spongiaeforme* var. *Tenue*, isolated from a litophytic sample collected in the Volcani Center, Bet Dagan, Israel.	Inhibit sea urchin embryos with ED_{100} of 10.8 μM.<br>--<br>Inhibit sea urchin embryos with ED_{100} of 9.0 μM.<br>Inhibit sea urchin embryos with ED_{100} of 19.1 μM.	[36]
74	Microcyclamide	Cyanobacterium *Microcystis aeruginosa* (NIES-298), obtained from the NIES collection (Microbial Culture Collection, the National Institute for Environmental Studies, Japan).	Moderate cytotoxicity against P388 murine leukemia cells.	[37]

75<br>76<br>77<br>78<br>79<br>80<br>81	Microcyclamide GL628<br>Microcyclamide GL614C<br>Microcyclamide GL582<br>Microcyclamide GL614A<br>Microcyclamide GL614B<br>Microcyclamide GL546A<br>Microcyclamide GL546B	A waterbloom of the cyanobacterium *Microcystis* sp., isolated from Gilboa reservoir, Valley of Armageddon, Israel.	Inhibit one solid tumor (A549-lung) and one leukemia (Molt-4) cell lines.	[38]
82	Cyanothecamide C	Marine cyanobacteria *Cyanothece* sp. PCC-7425, obtained from the Pasteur culture collection of cyanobacteria (Paris).	--	[39]
83	Cyclodidemnamide	Marine ascidian *Didemnum molly*, collected in the Philippine Islands.	Weakly cytotoxic toward human colon tumor cells. HCT-116, *in vitro*, with an ED_{50} of 16 µg/mL.	[40]
84<br>85	*cis, cis*-ceratospongamide<br>*trans, trans*-ceratospongamide	The Indonesian red alga *Ceratodictyon spongiosum* and symbiotic sponge *Sigmadocia symbiotica*.	--<br>Anti-inflammation; inhibit the expression of a human-sPLA2 promoter-based reporter by 90%.	[41]
86<br>87<br>88	Lissoclinamide 1<br>Lissoclinamide 2<br>Lissoclinamide 3	Tunicate *Lissoclinum patella*, collected in Iwayama Bay (near the Continental Hotel dock), Korror Island, Western Caroline Islands.	Borderline cytotoxicity in L1210 tissue culture assay.	[42]
89<br>90	Lissoclinamide 4<br>Lissoclinamide 5	Ascidian *Lissoclinum patella*, collected in Australia.	Cytotoxicity against human fibroblast, bladder carcinoma cell lines and normal lymphocytes; Marginal cytotoxicity against lymphocytic leukemia cells.	[43]
91	Lissoclinamide 6		Marginal cytotoxicity against lymphocytic leukemia cells with ED_{50} of 6.9 µg/mL.	
92<br>93	Lissoclinamide 7<br>Lissoclinamide 8		Cytotoxicity against human fibroblast, bladder carcinoma cell lines and normal lymphocytes.	[44]
94<br>95	Lissoclinamide 9<br>Lissoclinamide 10	An Indonesian collection of the ascidian *Lissoclinum Patella*.	--<br>--	[45]
96	Ulicyclamide	Ascidian *Lissoclinum patella*, collected from Palau, Western Caroline Islands.	Cytotoxic against cultured L1210 mouse leukemia cells.	[46]

97	Sanguinamide B	Nudibranch *Hexabranchus sanguineus*, collected from the Indo-Pacific.	Disrupted the twitching activity of *Pseudomonas aeruginosa.*	[47]
98<br>99<br>100	Myriastramide A<br>Myriastramide B<br>Myriastramide C	A Philippines marine sponge *Myriastra clavos.*	--	[48]
101<br>102	Haliclonamide A<br>Haliclonamide B	A Palauan marine sponge *Haliclona* sp.	--	[49]
103<br>104<br>105	Haliclonamide C<br>Haliclonamide D<br>Haliclonamide E		Repellent activity against the blue mussel *Mytilus edulis galloprovincialis.*	[50]
106<br>107	Perthamide J<br>Perthamide K	Marine sponge *T. swinhoe*i, collected at a depth of 22 m, on an isolated reef off the western coast of Malaita Island, Solomon Islands.	Anti-inflammatory.	[51]
108	Ascidiacyclamide	Ascidian, collected from Rodda Reef, Queensland, Australia.	Cytotoxicity.	[52]
109	Prepatellamide A	Ascidian *Lissoclinum patella*, collected in Indonesia.	Cytotoxicity against P388 murine leukemia cell lines observed in the crude extract with IC_{50} = ~5 μg/mL.	[53]
110	Prepatellamide B formate	Ascidian *Lissoclinum patella*, collected in Palau.	--	[54]
111	Patellamide C	Marine Tunicate *Lissoclinum patella*, collect from Palau of the Western Caroline Islands.	*In vitro* modulation of multidrug resistance in CEM/VBL100 cells; cytotoxicity against P388 murine leukemia cell lines.	[55]
112	Patellamide G	Ascidian *Lissoclinum patella*, collected in Pohnpei, Federated States of Micronesia.	Cytotoxicity against vinblastine-resistat CCRF-CEM human leukemic lymphoblasts; modest cytotoxicity in the NCI's 60 human tumor cell line panel (average LC_{50} values of 3 μM).	[56]
113	Patellamide A	Marine Tunicate *Lissoclinum Patella*, collect from Palau of the Western Caroline Islands.	Cytotoxicity against P388 murine leukemia cell lines.	[57]
114	Patellamide B		Modest general cytotoxicity in the NCI's 60 human tumor cell line panel (average LC_{50} values of 48 μM); *in vitro* modulation of multidrug resistance in CEM/VBL100 cells.	[55]
115	Patellamide D	Marine tunicate *Lissaclinum patella.*	Multidrug resistance in a human leukemic cell line.	[58]

116	Patellamide E	Ascidian *Lissoclinrrm patella*, collected at Pulau Salu, Singapore.	Mildly cytotoxic against human colon tumor cells *in vitro*.	[59]
117	Patellamide F	The colonial ascidian *Lissoclinrrm patella*, collected around Monte Bello Island, northwest Australia.	Modest general cytotoxicity in the NCI's 60 human tumor cell line panel (average LC_{50} values of 13 μM).	[60]
118	Kocurin	Marine-Derived Bacterium *Kocuria palustris* collected in Florida Keys, USA.	An excellent inhibitor of Methicillin-resistant *Staphylococcus aureus* (MRSA).	[42]
119	Mechercharstatin A	Marine-derived *Thermoactinomyces* sp. YM3-251, isolated from sea mud collected at Mecherchar in the Republic of Palau (North Pacific Ocean).	Strong antitumor activity.	[15]
120	Urukthapelstatin A	Marine-derived *Mechercharimyces asporophorigenens* YM11-542, isolated from a sediment sample collected from a marine lake in the northern part of Urukthapel Island in the Republic of Palau.	Potent cytotoxic activity against a human cancer cell line panel.	[61]
121	TP-1161	Marine *Nocardiopsis* sp., produced by a marine sediment-derived *Nocardiopsis*.	Strong antibacterial activity.	[62]
122	Wewakazole	Coral *Lyngbya majuscula*, collected from a shoreline growth of coral (< 1.5 m depth) in Wewak Bay, Papua New Guinea.	Inhibited cancer cell proliferation in a dose-dependent manner.	[63]
123	Wewakazole B	Cyanobacterium *Moorea producens* collected in the Red Sea.	Cytotoxic activity toward human MCF7 breast cancer cells (IC_{50} = 0.58 μM); human H460 lung cancer cells (IC_{50} = 1.0 μM).	[64]
124	Diazonamide A	Ascidian *Diazona chinensis*, collected from the ceilings of small caves along the northwest coast of Siquijor Island, Philippines.	Potent *in vitro* activity against HCT-116 human colon carcinoma and B-16 murine melanoma cancer cell lines, with IC_{50} values less than 15 ng/mL.	[65]
125	Diazonamide B		--	

126	Diazonamide C	Marine ascidian *Diazona* sp. collected in Indonesia.	Moderate cytotoxicity against a panel of three human tumor cell lines, including lung (A549), colon (HT29), and breast (MDA-MB-231).	[66]
127	Diazonamide D			
128	Diazonamide E			
129	Ulithiacyclamide	Ascidian *Lissoclinum patella*, collected from Palau, Western Caroline Islands.	IC_{50}'s of 35 ng/ml against the KB cell line.	[46]
130	Ulithiacyclamide B	*Lissoclinum patella* (SG-5 1) was collected from dead coral outside and north of Palikir Pass, Pohnpei at a depth of 2040 ft.	Exhibits an IC_{50}'s of 17 ng/ml against the KB cell line.	[67]
131	Ulithiacyclamide F	Ascidian *Lissoclinum patella*, collected in Australia.	Cytotoxicity against vinblastine-resistat CCRF-CEM human leukemic lymphoblasts.	[56]
132	Ulithiacyclamide G	Ascidian *Lissoclinum patella*, collected in Pohnpei, Federated States of Micronesia.	Cytotoxicity against vinblastine-resistat CCRF-CEM human leukemic lymphoblasts.	
133	Taumycin A	A Madagasca marine sponge *Fascaplysinopsis* sp.	Toxic to brine shrimp larvae; inhibited growth of the human UT-7 leukemic cell line.	[68]
134	Discokiolide A	Marine sponge *Discodermia kiiensis.*	Cytotoxic activities against P388, p388/ADM, B16-BL6, Lewis, Lu-99, HT-29 and CCD-19Lu.	[69]
135	Discokiolide B			
136	Discokiolide C			
137	Discokiolide D			
138	Phorboxazole A	The Indian Ocean marine sponge *Phorbas* sp.	High cytotoxic activity and induce cell growth inhibition (GI) in leukemia.	[70]
139	Phorboxazole B			
140	Hemi-phorboxazole A		A potent Michael acceptor.	[71]
141	Leiodolide A	Marine sponge *Leiodermatium,* collected at a depth of 720 feet near Uchelbeluu Reef in Palau.	Cytotoxic effects on human colon cancer HCT-116, HL-60, NCI-H52 and OVCAR-3 cell lines.	[72]
142	Leiodolide B			
143	Salarin C	Madagascan marine sponge *Fascaplysinopsis* sp. collected in Salary Bay, ca. 100 km north of Tulear.	Inhibit cell proliferation of human leukeamic cell lines UT-7 and K562 and the murine pro-B cell line Ba/F3.	[73]
144	Salarin F		Cytotoxicity against K562 and UT-7 human leukemia cells.	[74]
145	Salarin I			
146	Theonezolide A	An Okinawan marine sponge *Theonella* sp.	Cytotoxicity against murine lymphoma L1210 and human epidermoid carcinoma KB cells *in vitro*.	[75]
147	Theonezolide B			[76]

148	Theonezolide C			
149	Kabiramide I	A Thai marine sponge *Pachastrissa nux.*	Moderate antiplasmodial activity against *Plasmodium falciparum* K1.	[77]
150	Halishigamide B	An Okinawan marine sponge *Halichondria* sp.	Weak cytotoxicity against L1210 (IC_{50} 4.4 µg/mL) and KB cells (IC_{50} 7.5 µg/mL) and modest antifungal activity against *T. mentagrophytes* (MIC 25 µg/mL).	[78]
151	Mycalolide D	An Australian stony coral *Tubastrea faulkneri*.	Modest cytotoxicity against the NCI's 60-human tumor cell line panel.	[79]
152	Ulapualide A	Nudibranch *Hexabranchus sanguineus* eggs from the west shore of Oahu (Electric Beach).	Inhibit L1210 leukemia cell proliferation with IC_{50} 0.01 - 0.03 µg/mL and antifungi activity against *Candida albicans* .	[80]
153	Ulapualide B			
154	Ulapualide C		Submicromolar cytotoxicity against select NCI cell lines (768-0, DU-145, MDA-MB-231, and A549) with the most potent activity against MDA-MB-231 cells (IC_{50} 0.58 µM).	[81]
155	Ulapualide D		Antitumor.	
156	Ulapualide E		Antitumor.	
157	Miuramide A	Marine sponge *Mycale* sp., collected off Miura Peninsula.	Cytotoxicity against 3Y1 cells.	[82]
158	Miuramide B			
159	Mycalolide A	Marine sponge *Mycale* sp., collected from shallow waters (-2 - -5 m) in Gokasho Bay of the Kii Peninsula.	Antifungal effects on various pathogenic fungi and has cytotoxicity on B-16 melanoma cells with IC_{50} of 0.5 - 1.0 ng/mL.	[83]
160	Mycalolide B			
161	Mycalolide C			
162	38-hydroxymycalolide B	Marine sponge *Mycale magellanica*, collected at a depth of 15 m off Kumomi on the Izu Peninsula.	Cytotoxic against L1210 cells.	[84]
163	30-hydroxymycalolide A			
164	32-hydroxymycalolide A			
165	30,32-dihydroxymycalolide A	Marine sponge *Mycale izuensis*, collected in the Amakusa Islands 1700 km southwest of Tokyo.	Cytotoxic against HeLa cells with an IC_{50} value of 2.6 ng/mL.	[85]
166	Halichondramide	A Pacific marine sponge *Halichondria* sp.	Significant activity against *Candida albicans.*	[86]
167	*iso*-halichondramide	Marine sponge *Halichondria*, from Palau, Western Caroline Islands.	Antifungal activity and inhibit cell division in the fertilized sea urchin egg assay.	[87]
168	(19Z)-halichondramide	Marine sponge *Chondrosia corticata*, collected from Guam.	Significant cytotoxicity toward the human leukemia cell line K562.	[88]

169	Dihydrohalichondramid	Nudibranch *Hexabranchus sanguineus*, from Kwajalein Atoll, Marshall Islands.	Inhibited L1210 leukemia cell proliferation.	[87]
170	33-Methyltetrahydrohalichondramide	Nudibranch *Hexabranchus sanguineus*, collected from the Indo-Pacific.	Antifungal activity.	[47]
171<br>172<br>173	Jaspisamide A<br>Jaspisamide B<br>Jaspisamide C	Marine sponge *Jaspis* sp., collected by scuba off Ishigaki Island, Okinawa.	*In vitro* cytotoxicity against L1210 murine leukemia cells and KB human epidermoid carcinoma cells.	[89]
174	Halishigamide A	An Okinawan marine sponge *Halichondria* sp.	Potent cytotoxic activity against murine lymphoma L1210 and human epidermoid carcinoma KB cells (IC_{50} 0.0036 and 0.012 µg/mL, respectively) and antifungal activity against *Trichophyton mentagrophytes* (MIC 0.1 µg/mL).	[78]
175	Neohalichondramide	Marine sponge *Chondrosia corticata*, collected from Guam.	Exhibited significant cytotoxicity toward the human leukemia cell line K562.	[88]
176<br>177	Thiomycalolide A<br>Thiomycalolide B	Marine sponge *Mycale* sp., collected off the Kii Peninsula, 330 km southwest of Tokyo.	Highly cytotoxic against P388 murine leukemia cells.	[90]
178	Halichondramide acid	Marine sponge *Halichondria*, from Palau, Western Caroline Islands.	--	[87]
179	Mycalolide E	An Australian stony coral *Tubastrea faulkneri.*	Modest general cytotoxicity against the NCI's 60-human tumor cell line panel.	[83]
180	Kabiramide A	*Henabranchus* egg masses, collected at Kabira Bay, Ishigaki Island of the Ryukyus, and Hachijo Island of the Izu Archipelago.	Strongly active in the sea urchin egg assay; cytotoxic against L1210 cells.	[90]
181	Kabiramide D; 3-Carbamoyl	Nudibranch eggmasses, collected at Kabira Bay in Ishigaki-jima Island of the Ryukyus.	Marked antifungal activity.	[91]
182	Kabiramide E	*Henabranchus* egg masses, collected at Kabira Bay, Ishigaki Island of the Ryukyus, and Hachijo Island of the Izu Archipelago.	Strongly active in the sea urchin egg assay; cytotoxic against L1210 cells.	[90]

183	Tetrahydrohalichondramide	Nudibranch *Hexabranchus sanguineus*, from Kwajalein Atoll, Marshall Islands.	Antifungal activity; inhibit cell division in the fertilized sea urchin egg assay.	[87]
184	9-*O*-desmethylkbiramide B	Nudibranch *Hexabranchus sanguineus*, collected from the Indo-Pacific.	Antifungal activity.	[47]
185<br>186<br>187	Kabiramide B<br>Kabiramide C<br>Kabiramide D	Marine sponge *Pachastrissa nux*, collected from several other locations in the Gulf of Thailand.	Strong antimalarial and cytotoxic activities.	[92]
188	Kabiramide F	A Thai marine sponge *Pachastrissa nux.*	Cytotoxic against L1210 cells (IC_{50} 0.02 µg/mL).	[77]
189<br>190<br>191	Kabiramide G<br>Kabiramide J<br>Kabiramide K	Marine sponge *Pachastrissa nux*, collected from several other locations in the Gulf of Thailand.	Moderate to strong antimalarial; cytotoxic activities.	[92]
192	Kabiramide L		Moderate antiplasmodial activity against *Plasmodium falciparum* K1.	[93]
193	Neopeltolide	A deep-water sponge of the family *Neopeltidae*, collected off the north Jamaican coast.	Inhibitory activity against *C. albicans*; *in vitro* inhibit the proliferation of the A-549 human lung adenocarcinoma, the NCI-ADR-RES human ovarian sarcoma, and the P388 murine leukemia cell lines.	[94]
194	Leucascandrolide A	Marine sponge *Leucascandra caveolata*, collected along the east coats of New Caledonia, Coral Sea.	A strong cytotoxic activity on KB cells *in vitro*; inhibitory activity against *C. albicans.*	[95]
195	Enigmazole A	Marine sponge *Cinachyrella enigmatica*, isolated from a Papua New Guinea.	Cytotoxic to tumor NCI 60-cell line.	[96]
196	15-*O*-methylenigmazole A		--	
197	13-hydroxy-15-*O*-methylenigmazole A		--	
198	Calyculin A	Marine sponge *Discodermia calyx*, collected in the Gulf of Sagami at a depth of 5-15 m.	Significant activity in the starfish *Asterina pectinifera* and sea urchin *Hemicentrotus pulcherrimus*; highly cytotoxic against L1210 leukemia cells.	[97]
199	*Des-N*-methyl calyculin A	Marine sponge *Discodermia calyx*, collected by SCUBA at depths of 15 - 20 m off the Izu Peninsula.	Inhibited protein phosphatase 2A with an IC_{50} value of 49 nM.	[98]

200<br>201<br>202	Calyculin B<br>Calyculin C<br>Calyculin D	Marine sponge *Discodermia calyx*, collected in the Gulf of Sagami at a depth of 5 - 15 m.	Significant activity in the starfish *Asterina pectinifera* and sea urchin *Hemicentrotus pulcherrimus*; highly cytotoxic against L1210 leukemia cells.	[99]
203<br>204<br>205<br>206	Calyculin E<br>Calyculin F<br>Calyculin G<br>Calyculin H		Potent inhibitors of protein phosphatases 1 and 2A: effective doses for 50% inhibition of protein phosphatases 2A activity for these calyculins were 2.7 - 6.0 nM.	[100]
207	Dephosphonocalyculin A	Marine sponge *Discodermia calyx*,collected by SCUBA off the Izu Peninsula.	Inhibits protein phosphatases 1 and 2A with IC_{50} values of 3.0 and 8.2 nM, respectively.	[101]
208	Calyculinamide A	Marine sponge *Lamellomorpha strongylata*, collected by benthic dredging at -80 to -100 m along the top of the Mernoo Bank, within the Chatham Rise convergence zone.	Inhibited protein phosphatase 2A.	[102]
209	Calyculinamide F	Marine sponge *Discodermia calyx*, collected by SCUBA at depths of 15 - 20 m off the Izu Peninsula.	Inhibited protein phosphatase 2A.	[98]
210	Calyculinamide B	Marine sponge *Lamellomorpha strongylata*, collected by benthic dredging at -80 to -100 m along the top of the Mernoo Bank, within the Chatham Rise convergence zone.	A potent cell-growth inhibitor.	[102]
211<br>212	Clavosine A<br>Clavosine B	Marine sponge *Myriastra clavosa,* collected in 1993 from Chuuk, Federated States of Micronesia.	Potent cytotoxic in the National Cancer Institute's screening panel of 60 tumor cell lines; potent inhibitors of the type 1 and 2A serine/threonine protein phosphatases.	[103]
213	Clavosine C		--	
214	Geometricin A	Marine sponge *Luffariella geometrica*, collected at Heron Island's, Wistari Reef, Australia.	Moderately cytotoxic activity to tumor cell lines HM02 and HEPG2; antialgal activity.	[104]
215	Swinhoieamide A	Marine sponge *Theonella swinhoei*,collected by diving at a depth of 50 m near the coast of the Karkar Island, Papua New Guinea.	Insecticidal activity toward neonate larvae of the polyphagous pest insect *Spodoptera littoralis* when incorporated in an artificial diet offered to the larvae in a chronic feeding bioassay (ED_{50} 2.11 ppm,	[105]

			LD_{50} 2.98 ppm; inhibitory activity against *Candida albicans* and *Aspergillus fumigatus* (MIC 1.2 and 1.0 μg/mL, respectively).	
216	Calyculin J	Marine sponge *Discodermia calyx,* collected by SCUBA at depths of 15 - 20 m off the Izu Peninsula.	Inhibited protein phosphatase 2A.	[98]
217	Inthomycin B	*Streptomyces* sp. YB104, obtained from a deep-sea sediment sample collected from the South Atlantic Ocean at (19.57 °S, 11.97°W; −2699 m).	Antimicrobial activity.	[106]
218	*seco*-halichondramide	Marine sponge *Chondrosia corticata*, collected from Guam.	Weak cytotoxicity toward the human leukemia cell line K562.	[88]
219	Halishigamide C	An Okinawan marine sponge *Halichondria* sp.	Weak cytotoxicity against L1210 (IC_{50} 5.2 and 1.1 μg/mL) and KB cells (IC_{50} 6.5 and 1.8 μg/mL); modest antifungal activity against *T. mentagrophytes* (MIC 25 and 6.5 μg/mL).	[78]
220	Halishigamide D			
221	Kabiramide H	A Thai marine sponge *Pachastrissa nux*.	Cytotoxic.	[77]
222	Hennoxazole A	Marine sponge *Polyfibrospongia* sp., collectted from Agarihennazaki on the island of Miyako, Okinawa, Japan.	Antiviral; active against herpes simplex virus type 1 (IC_{50} 0.6 μg/mL); peripheral analgesic activity.	[107]
223	Hennoxazole B		Antiviral activity.	
224	Hennoxazole C			
225	Hennoxazole D			
226	Hennoxazole A; 4-Ac		--	[108]
227	Hennoxazole A; O2-De-Me (Hennoxazole E)		Cytotoxic against L1210.	
228	Hennoxazole F		Cytotoxic at a level of IC_{50} 2 μg/mL against L1210.	
229	Hennoxazole G		Cytotoxic against L1210.	
230	Antibiotic B-90063	Marine Bacterium *Blastobacter* sp. SANK 71894, isolated from sea water collected on the coast of Ojika Peninsula, Miyagi Pref., Japan.	An endothelin converting enzyme inhibitor.	[109]
231	R_1=C(=O)$(CH_2)_{14}CH_3$,	Marine sponge *Pachastrissa* sp. collected by	Strong active against *C. albicans.*	[110]

	$R_2=R_3=R_4=H$	scuba at Musha Archipelago, Republic of Djibouti, in a rocky slope at water deeper than 25 m.		
232	$R_4=C(=O)(CH_2)_{14}CH_3$, $R_1=R_2=R_3=H$			
233	$R_1=C(=O)(CH_2)_{12}CH(CH_3)_2$, $R_2=R_3=R_4=H$			
234	R_4=C(=O)(CH2)12CH(CH3)$_2$, $R_1=R_2=R_3=H$			
235	$R_1=C(=O)(CH_2)_{13}CH_3$, $R_2=R_3=R_4=H$			
236	$R_4=C(=O)(CH_2)_{13}CH_3$, $R_1=R_2=R_3=H$			
237	Bengazole A	Marine sponge of the *Choristida* order, obtained from new locations in the Benga Lagoon, Fiji.	Inhibition against two human tumor cell lines including colon, COLO-205 *in vitro*; anthelminthic activity; antifungal activity against *C. albicans.*	[111]
238	Bengazole B		Antifungal activity against *C. albicans.*	
239	Bengazole C	Marine sponge *Jaspis* sp., from the Great Barrier Reef.	Antifungal assay against *C. albicans.*	
240	Bengazole D			
241	Bengazole E			[112]
242	Bengazole F			
243	Bengazole G			
244	Bengazole B1			[113]
245	Bengazole Z		--	[114]
246	Bengazole C_2	Marine sponge *Choristid*, collected in Papua New Guinea.	Antifungal; cytotoxic; antiparasitic; tumor-promoting agents.	
247	Bengazole D_2			
248	Bengazole C_3			
249	Bengazole D_3			[115]
250	Bengazole C_4			
251	Bengazole D_4			
252	Bengazole C_6			

253	Digonazole	Marine sponge *Jaspis digonoxea*, collected in Sodwana Bay, South Africa.	Antifungal activity against *C. albicans.*	[116]
254	5-epi-Nakijinol C	Marine sponge *Dactylospongia metachromia*, collected at Ambon, Indonesia.	Strong inhibitory activity against ALK, FAK, IGF1-R, SRC, VEGF-R2, Aurora-B, MET wt, and NEK6 kinases.	[117]
255	(+)-5-epi-nakijinol D		--	
256	(+)-5-epi-nakijinol E	An Indonesian marine sponge *Smenospongia*.	Cytotoxic activity on colon cancer cells.	[118]
257	Nakijinol	Marine sponge of the family *Spongiidae*, collected off Nakijin, Okinawa Island.	--	[119]
258	(-)-Nakijinol E	An Indonesian marine sponge *Smenospongia*.	Cytotoxic activity on colon cancer cells.	[118]
259	Nakijinol B	Marine sponge *Dactylospon1gia elegans*, collected from Pugh Shoal, northeast of Truant Island, NT.	Cytotoxicity against a panel of human tumor cell lines (SF-268, H460, MCF-7, and HT-29) and a normal mammalian cell line (CHO-K1).	[120]
260	Nakijinol B diacetate			
261	Nakijinol E	Marine sponge *Hyrtios* sp., collected off Yongxing Island in the South China Sea.	Anti-inflammatory.	[121]
262	Nakijinol F		Antitumor.	
263	Nakijinol G		PTP1B inhibitory activity with an IC_{50} value of 4.8 μM.	
264	Nocarbenzoxazole A	A saltmarsh soil sample, *Nocardiopsis lucentensis* DSM 44048.	Antimicrobial activity; antibiotic activity.	[122]
265	Nocarbenzoxazole B		Antimicrobial activity; antibiotic activity.	
266	Nocarbenzoxazole C		Antimicrobial activity; antibiotic activity.	
267	Nocarbenzoxazole D		Antimicrobial activity; antibiotic activity.	
268	Nocarbenzoxazole E		Antimicrobial activity; antibiotic activity.	
269	Nocarbenzoxazole F		Anti-inflammatory.	
270	Nocarbenzoxazole G		Selective activity against HepG2 and HeLa cell lines.	
271	Coixol	Marine sponge *Oceanapia* sp., collected at a depth of 20 ft by skin diving near Mandapam coast (N 17°, E 83°) India.	Active against brine shrimp assay.	[123]
272	Homopseudopteroxazole	Sea plume *Pseudopterogorgia elisabethae*,	A strong growth inhibitor of *Mycobacterium tuberculosis* H37Rv.	[124]

		collected by scuba during an expedition to San Andre's Island, Colombia.		
273<br>274	Pseudopteroxazole<br>*seco*-pseudopteroxazole	Sea whip *Pseudopterogorgia elisabethae*, collected near San Andre's Island, Colombia.	Effect potent inhibitory activity (97%) against *M. tuberculosis* H37Rv at a concentration of 12.5 μg/mL.<br>Inhibited 66% of mycobacterial growth.	[125]
275	Ileabethoxazole	Sea whip *Pseudopterogorgia elisabethae* removed by scuba from their natural habitat at depths of 25 - 30 m near the Island of Providencia (Old Providence), Colombia.	92% inhibition of *Mycobacterium tuberculosis* (H37Rv) at the concentration range of 128 - 64 μg/ mL .	[126]
276	Oxazocurcuphenol	Soft coral *Pseudopterogorgia rigida*, collected by SCUBA diving southeast of Lighthouse Point in Eleuthera island in the Bahamas, at a depth of 20 - 30 m.	--	[130]
277	Citharoxazole	Marine sponge *Latrunculia* (*Biannulata*) *citharistae*, found at depths around 130 - 150 m in the western Mediterranean sea.	--	[127]
278	Herqueioxazole	Marine-derived fungus *Penicillium* sp. from a marine sediment.	Marginally active against the lung carcinoma A549 cell line.	[128]
279<br>280	Nakijinamine C<br>Nakijinamine E	An Okinawan marine sponge *Suberites* sp.	Antifungal activity against *Aspergillis niger* with MIC values of 16 μg/mL .	[129]
281	Caboxamycin	*Streptomyces* sp. NTK 937, isolated from an Atlantic Ocean deep-sea sediment core.	Antibiotic activity; a moderate growth inhibitory activity towards AGS, HepG2 and MCF7.	[130]
282	Hamigeran M	Marine sponge *Hamigera tarangaensis*, collected from Cavalli Island, New Zealand.	The high efficacy against HL-60 promyelocytic leukemia cell line.	[131]
283<br>284	Citreamicin θA<br>Citreamicin θB	Marine-derived *Streptomyces* sp., isolated from the coastal water of the red sea by the side of a fish market near Jeddah.	Antibacterial activity against *Staphylococcus haemolyticus*, *Staphylococcus aureus*, and *Bacillus subtillis* with MIC of 0.25 μg/mL.	[132]
285	Ergosinine	Sea slug *Pleurobranchus forskalii*, collected off Ishigaki Island, Japan.	--	[133]

References

1. N'Diaye, I.; Guella, G.; Chiasera, G.; Mancini, I.; Pietra, F., Almazole A and almazole B, unusual marine alkaloids of an unidentified red seaweed of the family delesseriaceae from the coasts of Senegal. *Tetrahedron Lett.* **1994**, *35*, 4827-4830. [CrossRef]
2. Guella, G.; Mancini, I.; Pietra, F., Almazole C, a new indole alkaloid bearing an unusually 2,5-disubstituted oxazole moiety, and its putative biogenetic peptidic precursors, from a senegalese delesseriacean seaweed. *Helvetica Chimica Acta.* **1994**, *77*, 1999-2006. [CrossRef]
3. N'Diaye, I.; Guella, G.; Mancini, I.; Pietra, F., Almazole D, a new type of antibacterial 2,5-disubstituted oxazolic dipeptide from a red alga of the coast of Senegal. *Tetrahedron Lett.*, **1996**, *37*, 3049-3050. [CrossRef]
4. Shin, H.J.; Jeong, H.S.; Lee, H.S.; Park, S.K.; Kim, H.M.; Kwon, H.J., Isolation and structure determination of streptochlorin, an antiproliferative agent from a marine-derived *Streptomyces* sp. 04DH110. *J. Microbiol. Biotechnol.* **2007**, *17*, 1403-1406. [CrossRef]
5. Takahashi, S.; Matsunaga, T.; Hasegawa, C.; Saito, H.; Fujita, D.; Kiuchi, F.; Tsuda, Y., Martefragin A, a novel indole alkaloid isolated from red alga, inhibits lipid peroxidation. *Chem. Pharm. Bull.* **1998**, *46*, 1527-1529. [CrossRef]
6. Guella, G.; N'Diaye, I.; Fofana, M.; Mancini, I., Isolation, synthesis and photochemical properties of almazolone, a new indole alkaloid from a red alga of Senegal. *Tetrahedron* **2006**, *62*, 1165-1170. [CrossRef]
7. Schneider, Y.; Jenssen, M.; Isaksson, J.; Hansen, K.O.; Andersen, J.H.; Hansen, E.H., Bioactivity of serratiochelin A, a siderophore isolated from a co-culture of *Serratia* sp. and *Shewanella* sp. *Microorganisms* **2020**, *8*, 1-17. [CrossRef]
8. Motohashi, K.; Takagi, M.; Shin-Ya, K., Tetrapeptides possessing a unique skeleton, JBIR-34 and JBIR-35, isolated from a sponge-derived actinomycete, *Streptomyces* sp. Sp080513GE-23. *J. Nat. Prod.* **2010**, *73*, 226-228. [CrossRef]
9. Nielsen, A.; Mansson, M.; Wietz, M.; Varming, A.N.; Phipps, R.K.; Larsen, T.O.; Gram, L.; Ingmer, H., Nigribactin, a novel siderophore from Vibrio nigripulchritudo, modulates *Staphylococcus aureus* virulence gene expression. *Mar. Drugs.* **2012**, *10*, 2584-2595. [CrossRef]
10. Rudi, A.; Stein, Z.; Green, S.; Goldberg, I.; Kashman, Y.; Benayahu, Y.; Schleyer, M., Phorbazoles A-D, novel chlorinated phenylpyrrolyloxazoles from the marine sponge *Phorbas aff. clathrata*. *Tetrahedron Lett.* **1994**, *35*, 2589-2592. [CrossRef]
11. Nuzzo, G.; Ciavatta, M.L.; Kiss, R.; Mathieu, V.; Leclercqz, H.; Manzo, E.; Villani, G.; Mollo, E.; Lefranc, F.; D'Souza, L.; Gavagnin, M.; Cimino, G., Chemistry of the nudibranch *Aldisa andersoni*: structure and biological activity of phorbazole metabolites. *Mar. Drugs* **2012**, *10*, 1799-1811. [CrossRef]
12. Oku, N.; Adachi, K.; Matsuda, S.; Kasai, H.; Takatsuki, A.; Shizuri, Y., Ariakemicins A and B, novel polyketide-peptide antibiotics from a marine gliding bacterium of the genus *Rapidithrix*. *Org. Lett.* **2008**, *10*, 2481-2484. [CrossRef]
13. Hanssen, K.O.; Schuler, B.; Williams, A.J.; Demissie, T.B.; Hansen, E.; Andersen, J.H.; Svenson, J.; Blinov, K.; Repisky, M.; Mohn, F.; Meyer, G.; Svendsen, J.S.; Ruud, K.; Elyashberg, M.; Gross, L.; Jaspars, M.; Isaksson, J., A combined atomic force microscopy and computational approach for the structural elucidation of breitfussin A and B: highly modified halogenated dipeptides from *Thuiaria breitfussi*. *Angew Chem. Int. Ed. Engl.* **2012**, *51*, 12238-12241. [CrossRef]

14. Hansen, K.O.; Andersen, J.H.; Bayer, A.; Pandey, S.K.; Lorentzen, M.; Jorgensen, K.B.; Sydnes, M.O.; Guttormsen, Y.; Baumann, M.; Koch, U.; Klebl, B.; Eickhoff, J.; Haug, B.E.; Isaksson, J.; Hansen, E.H., Kinase chemodiversity from the arctic: the breitfussins. *J. Med. Chem.* **2019**, *62*, (22), 10167-10181. [CrossRef]
15. [15] Kanoh, K.; Matsuo, Y.; Adachi, K.; Imagawa, H.; Nishizawa, M.; Shizuri, Y., Mechercharmycins A and B, cytotoxic substances from marine-derived *Thermoactinomyces* sp. YM3-251. *J. Antibiot.* **2005**, *58*, 289-292. [CrossRef]
16. Nett, M.; Erol, O.; Kehraus, S.; Kock, M.; Krick, A.; Eguereva, E.; Neu, E.; Konig, G.M., Siphonazole, an unusual metabolite from *Herpetosiphon* sp. *Angew Chem. Int. Ed. Engl.* **2006**, *45*, 3863-3867. [CrossRef]
17. Fukuhara, K.; Takada, K.; Okada, S.; Matsunaga, S., Nazumazoles A-C, cyclic pentapeptides dimerized through a disulfide bond from the marine sponge *Theonella swinhoei*. *Org. Lett.* **2015**, *17*, 2646-2648. [CrossRef]
18. Fukuhara, K.; Takada, K.; Okada, S.; Matsunaga, S., Nazumazoles D-F, cyclic pentapeptides that inhibit chymotrypsin, from the marine sponge *Theonella swinhoei*. *J. Nat. Prod.* **2016**, *79*, 1694-1697. [CrossRef]
19. Nobuhiro Fusetani, T.S., and Shigeki Matsunaga, Orbiculamide A: a novel cytotoxic cyclic peptide from a marine sponge *Theonella* sp. *J. Am. Chem. Soc.* **1991**, *113*, 7811-7812. [CrossRef]
20. Kobayashi, J.; Itagaki, F.; Shigemori, H.; Ishibashi, M.; Takahashi, K.; Ogura, M.; Nagasawa, S.; Nakamura, T.; Hirota, H., Keramamides B-D, novel peptides from the Okinawan marine sponge *Theonella* sp. *J. Am. Chem. Soc.* **1991**, *113*, 7812-7813. [CrossRef]
21. Kobayashi, J.i.; Itagaki, F.; Shigemori, I.; Takao, T.; Shimonishi, Y., Keramamides E, G, H, and J, new cyclic peptides containing an oxazole or a thiazole ring from a *Theonella* sponge. *Tetrahedron* **1995**, *51*, 2525-2532. [CrossRef]
22. Tsuda, M.; Ishiyama, H.; Masuko, K.; Takao, T.; Shimonishi, Y.; Kobayashi, J.i., Keramamides M and N, two new cyclic peptides with a sulfate ester from *Theonella* sponge. *Tetrahedon* **1999**, *55*, 12543-12548. [CrossRef]
23. Gunasekera, S.P.; Pomponi, S.A.; McCarthy, P.J., Discobahamins A and B, new peptides from the Bahamian deep water marine sponge *Discodermia* sp. *J. Nat. Prod.* **1994**, *57*, 79-83. [CrossRef]
24. Degnan, B.M.; Hawkins, C.J.; Lavin, M.F.; McCaffrey, E.J.; Parry, D.L.; Watters, D.J., Novel cytotoxic compounds from the ascidian *Lissoclinum bistratum*. *J. Med. Chem.* **1989**, *32*, 1354-1359. [CrossRef]
25. Foster, M.P.; Concepcion, G.P.; Caraan, G.B.; Ireland, C.M., Bistratamides C and D. Two new oxazole-containing cyclic hexapeptides isolated from a Philippine *Lissoclinum bistratum* ascidian. *J. Org. Chem.* **1992**, *57*, 6671-6675. [CrossRef]
26. Perez, L.J.; Faulkner, D.J., Bistratamides E-J, modified cyclic hexapeptides from the Philippines ascidian *Lissoclinum bistratum*. *J. Nat. Prod.* **2003**, *66*, 247-250. [CrossRef]
27. Urda, C.; Fernandez, R.; Rodriguez, J.; Perez, M.; Jimenez, C.; Cuevas, C., Bistratamides M and N, oxazole-thiazole containing cyclic hexapeptides isolated from *Lissoclinum bistratum* interaction of Zinc (II) with bistratamide K. *Mar. Drugs.* **2017**, *15*, 1-11. [CrossRef]
28. Prinsep, M.R.; Moore, R.E.; Levine, I.A.; Patterson, G.M., Westiellamide, a bistratamide-related cyclic peptide from the blue-green alga *Westiellopsis prolifica*. *J. Nat. Prod.* **1992**, *55*, 140-142. [CrossRef]

29. Ogino, J.; Moore, R.E.; Patterson, G.M.; Smith, C.D., Dendroamides, new cyclic hexapeptides from a blue-green alga. Multidrug-resistance reversing activity of dendroamide A. *J. Nat. Prod.* **1996**, *59*, 581-586. [CrossRef]
30. Rudi, A.; Chill, L.; Aknin, M.; Kashman, Y., Didmolamide A and B, two new cyclic hexapeptides from the marine ascidian *Didemnum molle*. *J. Nat. Prod.* **2003**, *66*, 575-577. [CrossRef]
31. Ojika, M.; Nemoto, T.; Nakamura, M.; Yamada, K., Dolastatin E, a new cyclic hexapeptide isolated from the sea hare *Dolabella auricularia*. *Tetrahedron Lett.* **1995**, *36*, 5057-5058. [CrossRef]
32. Sone, H.; Kigoshi, H.; Yamada, K., Isolation and stereostructure of dolastatin I, a cytotoxic cyclic hexapeptide from the Japanese sea hare *Dolabella auricularia*. *Tetrahedron* **1997**, *53*, 8149-8154. [CrossRef]
33. Kehraus, S.; Konig, G.M.; Wright, A.D.; Woerheide, G., Leucamide A: a new cytotoxic heptapeptide from the Australian sponge *Leucetta microraphis*. *J. Org. Chem.* **2002**, *67*, 4989-4992. [CrossRef]
34. Rudi, A.; Aknin, M.; Gaydou, E.M.; Kashman, Y., Four new cytotoxic cyclic hexa- and heptapeptides from the marine ascidian *Didemnum molle*. *Tetrahedron* **1998**, *54*, 13203-13210. [CrossRef]
35. Linington, R.G.; Gonzalez, J.; Urena, L.D.; Romero, L.I.; Ortega-Barria, E.; Gerwick, W.H., Venturamides A and B: antimalarial constituents of the panamanian marine cyanobacterium *Oscillatoria* sp. *J. Nat. Prod.* **2007**, *70*, 397-401. [CrossRef]
36. Banker, R.; Carmeli, S., Tenuecyclamides A-D, cyclic hexapeptides from the cyanobacterium *Nostoc spongiaeforme* var. *tenue*. *J. Nat. Prod.* **1998**, *61*, 1248-1251. [CrossRef]
37. shida, K.; Nakagawa, H.; Murakami, M., Microcyclamide, a cytotoxic cyclic hexapeptide from the cyanobacterium *Microcystis aeruginosa*. *J. Nat. Prod.,* **2000**, *63*, (9), 1315-1317. [CrossRef]
38. Raveh, A.; Moshe, S.; Evron, Z.; Flescher, E.; Carmeli, S., Novel thiazole and oxazole containing cyclic hexapeptides from a waterbloom of the cyanobacterium *Microcystis* sp. *Tetrahedron,* **2010**, *66*, 2705-2712. [CrossRef]
39. Houssen, W.E.; Koehnke, J.; Zollman, D.; Vendome, J.; Raab, A.; Smith, M.C.; Naismith, J.H.; Jaspars, M., The discovery of new cyanobactins from *Cyanothece* PCC 7425 defines a new signature for processing of patellamides. *Chembiochem.,* **2012**, *13*, (18), 2683-2689. [CrossRef]
40. Toske, S.G.; Fenical, W., Cyclodidemnamide: a new cyclic heptapeptide from the marine ascidian *Didemnum Molle*. *Tetrahedron Lett.* **1995**, *36*, 8355-8358. [CrossRef]
41. Tan, L.T.; Williamson, R.T.; Gerwick, W.H.; Watts, K.S.; McGough, K.; Jacobs, R., *Cis,cis*- and *trans,trans*-ceratospongamide, new bioactive cyclic heptapeptides from the Indonesian red alga *Ceratodictyon spongiosum* and symbiotic sponge *Sigmadocia symbiotica*. *J. Org. Chem.* **2000**, *65*, 419-425. [CrossRef]
42. Wasylyk J. M.; Biskupiak J. E.; Costello C. E.; Ireland C. M., Cyclic peptide structures from the tunicate *Lissoclinum patella* by FAB mass spectrometry. *J. Org. Chem.* **1983** *48*, 4445-4449. [CrossRef]
43. Schmitz, F.J.; Ksebati, M.B.; Chang, J.S.; Wang, J.L.; Hossain, M.B.; Van der Helm, D.; Engel, M.H.; Serban, A.; Silfer, J.A., Cyclic peptides from the ascidian *Lissoclinum patella*: conformational analysis of patellamide D by x-ray analysis and molecular modeling. *J. Org. Chem.* **2002**, *54*, 3463-3472. [CrossRef]

44. Hawkins, C.J.; Lavin, M.F.; Marshall, K.A.; van den Brenk, A.L.; Watters, D.J., Structure-activity relationships of the lissoclinamides: cytotoxic cyclic peptides from the ascidian *Lissoclinum patella*. *J. Med. Chem.* **1990**, *33*, 1634-1638. [CrossRef]
45. Morris, L.A.; Jantina Kettenes van den Bosch, J.; Versluis, K.; Thompson, G.S.; Jaspars, M., Structure determination and MSn analysis of two new lissoclinamides isolated from the Indo–Pacific ascidian *Lissoclinum patella*: NOE restrained molecular dynamics confirms the absolute stereochemistry derived by degradative methods. *Tetrahedron* **2000**, *56*, 8345-8353. [CrossRef]
46. Ireland, C.; Scheuer, P.J., Ulicyclamide and ulithiacyclamide, two new small peptides from a marine tunicate. *J. Am. Chem. Soc.* **1980**, *102*, 5688-5691. [CrossRef]
47. Dalisay, D.S.; Rogers, E.W.; Edison, A.S.; Molinski, T.F., Structure elucidation at the nanomole scale. 1. Trisoxazole macrolides and thiazole-containing cyclic peptides from the nudibranch *Hexabranchus sanguineus*. *J. Nat. Prod.* **2009**, *72*, 732-738. [CrossRef]
48. Erickson, K.L.; Gustafson, K.R.; Milanowski, D.J.; Pannell, L.K.; Klose, J.R.; Boyd, M.R., Myriastramides A–C, new modified cyclic peptides from the Philippines marine sponge *Myriastra clavosa*. *Tetrahedron* **2003**, *59*, 10231-10238.[CrossRef]
49. Guan, L.L.; Sera, Y.; Adachi, K.; Nishida, F.; Shizuri, Y., Isolation and evaluation of nonsiderophore cyclic peptides from marine sponges. *Biochem. Biophys. Res. Commun.* **2001**, *283*, 976-981. [CrossRef]
50. Sera, Y.; Adachi, K.; Fujii, K.; Shizuri, Y., Isolation of haliclonamides: new peptides as antifouling substances from a marine sponge species, *Haliclona*. *Mar. Biotechnol (NY).* **2002**, *4*, 441-446. [CrossRef]
51. Festa, C.; De Marino, S.; D'Auria, M.V.; Monti, M.C.; Bucci, M.; Vellecco, V.; Debitus, C.; Zampella, A., Anti-inflammatory cyclopeptides from the marine sponge *Theonella swinhoei*. *Tetrahedron* **2012**, *68*, 2851-2857. [CrossRef]
52. Hamamoto, Y.; Endo, M.; Nakagawa, M.; Nakanishi, T.; Mizukawa, K., A new cyclic peptide, ascidiacyclamide, isolated from ascidian. *J. Chem. Soc. Chem Commum.* **1983**, *5*, 323-324. [CrossRef]
53. Fu, X.; Su, J.Y.; Zeng, L.M., Prepatellamide A, a new cyclic peptide from the ascidian *Lissoclinum patella*. *Sci. China Ser. B.* **2000**, *43*, 643-648. [CrossRef]
54. Sesin, D.F.; Gaskell, S.J.; Ireland, C.M., The chemistry of *Lissoclinum Patella*. *Bull. Soc. Chim. Belq.* **1986**, *95*, 853-867. [CrossRef]
55. Ireland, C.M.; Durso, A.R.; Newman, R.A.; Hacker, M.P., Antineoplastic cyclic peptides from the marine tunicate *Lissoclinum patella*. *J. Org Chem.* **2002**, *47*, 1807-1811. [CrossRef]
56. Fu, X.; Do, T.; Schmitz, F.J.; Andrusevich, V.; Engel, M.H., New cyclic peptides from the ascidian *Lissoclinum patella*. *J. Nat. Prod.*, **1998**, *61*, (12), 1547-1551. [CrossRef]
57. In, Y.; Doi, M.; Inoue, M.; Ishida, T.; Hamada, Y.; Shioiri, T., Patellamide A, a cytotoxic cyclic peptide from the ascidian *Lissoclinum patella*. *Acta. Crystallogr C.*, **1994**, *50*, (3), 432-434. [CrossRef]
58. Williams, A.B.; Jacobs, R.S., A marine natural product, patellamide D, reverses multidrug resistance in a human leukemic cell line. *Cancer Lett.* **1993**, *71*, 97-102.[CrossRef]
59. McDonald, L.A.; Ireland, C.M., Patellamide E: a new cyclic peptide from the ascidian *Lissoclinum patella*. *J. Nat. Prod.* **1992**, *55*, 376-379. [CrossRef]

60. Rashid, M.A.; Gustafson, K.R.; Cardellina, J.H.; Boyd, M.R., Patellamide F, A new cytotoxic cyclic peptide from the colonial ascidian *Lissoclinum patella*. *J. Nat. Prod.* **1995**, *58*, 594-597.[CrossRef]
61. Matsuo, Y.; Kanoh, K.; Yamori, T.; Kasai, H.; Katsuta, A.; Adachi, K.; Shin-Ya, K.; Shizuri, Y., Urukthapelstatin A, a novel cytotoxic substance from marine-derived *Mechercharimyces asporophorigenens* YM11-542. I. Fermentation, isolation and biological activities. *J. Antibiot* **2007**, *60*, 251-255. [CrossRef]
62. Engelhardt, K.; Degnes, K.F.; Kemmler, M.; Bredholt, H.; Fjaervik, E.; Klinkenberg, G.; Sletta, H.; Ellingsen, T.E.; Zotchev, S.B., Production of a new thiopeptide antibiotic, TP-1161, by a marine *Nocardiopsis* species. *Appl. Environ. Microbiol.* **2010**, *76*, 4969-4976. [CrossRef]
63. Nogle, L.M.; Marquez, B.L.; Gerwick, W.H., Wewakazole, a novel cyclic dodecapeptide from a Papua new guinea *Lyngbya majuscula*. *Org. Lett.* **2003**, *5*, 3-6. [CrossRef]
64. Lopez, J.A.; Al-Lihaibi, S.S.; Alarif, W.M.; Abdel-Lateff, A.; Nogata, Y.; Washio, K.; Morikawa, M.; Okino, T., Wewakazole B, a cytotoxic cyanobactin from the cyanobacterium *Moorea producens* collected in the red sea. *J. Nat. Prod.* **2016**, *79*, 1213-1218. [CrossRef]
65. Lindquist, N.; Fenical, W.; Van Duyne, G.D.; Clardy, J., Isolation and structure determination of diazonamides A and B, unusual cytotoxic metabolites from the marine ascidian *Diazona chinensis*. *J. Am. Chem. Soc.* **1991**, *113*, 2303-2304. [CrossRef]
66. Fernández, R.; Martín, M.J.; Rodríguez-Acebes, R.; Reyes, F.; Francesch, A.; Cuevas, C., Diazonamides C–E, new cytotoxic metabolites from the ascidian *Diazona* sp. *Tetrahedron Lett.* **2008**, *49*, 2283-2285. [CrossRef]
67. Williams, D.E.; Moore, R.E.; Paul, V.J., The structure of ulithiacyclamide B. Antitumor evaluation of cyclic peptides and macrolides from *Lissoclinum patella*. *J. Nat. Prod.* **1989**, *52*, 732-739. [CrossRef]
68. Bishara, A.; Rudi, A.; Aknin, M.; Neumann, D.; Ben-Califa, N.; Kashman, Y., Taumycins A and B, two bioactive lipodepsipeptides from the Madagascar sponge *Fascaplysinopsis* sp. *Org. Lett.* **2008**, *10*, 4307-4309. [CrossRef]
69. Tada, H.; Tozyo, T.; Terui, Y.; Hayashi, F., Discokiolides. Cytotoxic cyclic depsipeptides from the marine sponge *Discodermia kiiensis*. *Chem. Lett.* **1992**, 431-434. [CrossRef]
70. Searle, P.A.; Molinski, T.F.; Brzezinski, L.J.; Leahy, J.W., Absolute configuration of phorboxazoles A and B from the marine sponge *Phorbas* sp. 1. Macrolide and hemiketal rings. *J. Am. Chem. Soc.* **1996**, *118*, 9422-9423.[CrossRef]
71. Dalisay, D.S.; Molinski, T.F., Structure elucidation at the nanomole scale. 2. Hemi-phorboxazole A from *Phorbas* sp. *Org. Lett.* **2009**, *11*, 1967-1970. [CrossRef]
72. Sandler, J.S.; Colin, P.L.; Kelly, M.; Fenical, W., Cytotoxic macrolides from a new species of the deep-water marine sponge *Leiodermatium*. *J. Org. Chem.* **2006**, *71*, 7245-7251. [CrossRef]
73. Bishara, A.; Rudi, A.; Aknin, M.; Neumann, D.; Ben-Califa, N.; Kashman, Y., Salarin C, a new cytotoxic sponge-derived nitrogenous macrolide. *Tetrahedron Lett.* **2008**, *49*, 4355-4358. [CrossRef]
74. Bishara, A.; Rudi, A.; Aknin, M.; Neumann, D.; Ben-Califa, N.; Kashman, Y., Salarins D-J, seven new nitrogenous macrolides from the madagascar sponge *Fascaplysinopsis* sp. *Tetrahedron* **2010**, *66*, 4339-4345. [CrossRef]
75. Kobayashi, J.; Kondo, K.; Ishibashi, M.; Walchli, M.R.; Nakamura, T., Theonezolide A: a novel polyketide natural product from the Okinawan marine sponge *Theonella* sp. *J. Am. Chem. Soc.* **1993**, *115*, 6661-6665. [CrossRef]

76. Kondo, K.; Ishibashi, M.; Kobayashi, J.i., Isolation and structures of theonezolides B and C from the Okinawan marine sponge *Theonella* sp. *Tetrahedron* **1994**, *50*, 8355-8362. [CrossRef]
77. Petchprayoon, C.; Asato, Y.; Higa, T.; Garcia-Fernandez, L.F.; Pedpradab, S.; Marriott, G.; Suwanborirux, K.; Tanaka, J., Four new kabiramides from the Thai sponge, *Pachastrissa nux*. *Heterocycles* **2006**, *69*, 447-+. [CrossRef]
78. Kobayashi, J.i.; Tsuda, M.; Fuse, H.; Sasaki, T.; Mikami, Y., Halishigamides A-D, new cytotoxic oxazole-containing metabolites from Okinawan sponge *Halichondria* sp. *J. Nat. Prod.* **1997**, *60*, 150-154. [CrossRef]
79. Rashid, M.A.; Gustafson, K.R.; Cardellina II, J.H.; Boyd, M.R., Mycalolides D and E, new cytotoxic macrolides from a collection of the stony coral *Tubastrea faulkneri*. *J. Nat. Prod.* **1995**, *58*, 1120-1125. [CrossRef]
80. Roesener, J.A.; Scheuer, P.J., Ulapualide A and B, extraordinary antitumor macrolides from nudibranch eggmasses. *J. Am Chem Soc.* **1986**, *108*, 846-847. [CrossRef]
81. Parrish, S.M.; Yoshida, W.; Yang, B.; Williams, P.G., Ulapualides C-E isolated from a Hawaiian *Hexabranchus sanguineus* egg mass. *J. Nat. Prod.* **2017**, *80*, 726-730. [CrossRef]
82. Suo, R.; Takada, K.; Kohtsuka, H.; Ise, Y.; Okada, S.; Matsunaga, S., Miuramides A and B, trisoxazole macrolides from a *Mycale* sp. marine sponge that induce a protrusion phenotype in cultured mammalian cells. *J. Nat Prod.* **2018**, *81*, 1108-1112. [CrossRef]
83. Fusetani, N.; Yasumuro, K.; Matsunaga, S.; Hashimoto, K., Mycalolides A- C, hybrid macrolides of ulapualides and halichondramide, from a sponge of the genus *Mycale*. *Tetrahedron Lett.* **1989**, *30*, 2809-2812. [CrossRef]
84. Matsunaga, S.; Sugawara, T.; Fusetani, N., New mycalolides from the marine sponge *Mycale magellanica* and their interconversion. *J. Nat. Prod.* **1998**, *61*, 1164-1167. [CrossRef]
85. Phuwapraisirisan, P.; Matsunaga, S.; van Soest, R.W.; Fusetani, N., Isolation of a new mycalolide from the marine sponge *Mycale izuensis*. *J. Nat. Prod.* **2002**, *65*, 942-943. [CrossRef]
86. Kernan, M.R.; Faulkner, D.J., Halichondramide, an antifungal macrolide from the sponge *Halichondria* sp. *Tetrahedron Lett.* **1987**, *28*, 2809-2812. [CrossRef]
87. Kernan, M.R.; Molinski, T.F.; Faulkner, D.J., Macrocyclic antifungal metabolites from the Spanish dancer nudibranch *Hexabranchus sanguineus* and sponges of the genus *Halichondria*. *J. Org. Chem.* **1988**, *53*, 5014-5020. [CrossRef]
88. Shin, J.; Lee, H.S.; Kim, J.Y.; Shin, H.J.; Ahn, J.W.; Paul, V.J., New macrolides from the sponge *Chondrosia corticata*. *J. Nat Prod.* **2004**, *67*, 1889-1892. [CrossRef]
89. Kobayashi, J.i.; Murata, O.; Shigemori, H.; Sasaki, T., Jaspisamides A-C, new cytotoxic macrolides from the Okinawan sponge *Jaspis* sp. *J. Nat Prod.* **1993**, *56*, 787-791. [CrossRef]
90. Matsunaga, S., Further kabiramides and halichondramides, cytotoxic macrolides embracing trisoxazole, from the *Hexabranch* us Egg Masses. *J. Org. Chem.* **1989**, *54*, 1360-1363. [CrossRef]
91. Matsunaga, S.; Fusetani, N.; Hashimoto, K., Kabiramide C, a novel antifungal macrolide from nudibranch eggmasses. *J. Am. Chem. Soc.* **1986**, *108*, 847-849. [CrossRef]

92. Sirirak, T.; Kittiwisut, S.; Janma, C.; Yuenyongsawad, S.; Suwanborirux, K.; Plubrukarn, A., Kabiramides J and K, trisoxazole macrolides from the sponge *Pachastrissa nux*. *J. Nat. Prod.* **2011**, *74*, 1288-1292. [CrossRef]
93. Sirirak, T.; Brecker, L.; Plubrukarn, A., Kabiramide L, a new antiplasmodial trisoxazole macrolide from the sponge *Pachastrissa nux*. *Nat. Prod. Res.*, **2013**, *27*, (13), 1213-1219. [CrossRef]
94. Wright, A.E.; Botelho, J.C.; Guzman, E.; Harmody, D.; Linley, P.; McCarthy, P.J.; Pitts, T.P.; Pomponi, S.A.; Reed, J.K., Neopeltolide, a macrolide from a lithistid sponge of the family *Neopeltidae*. *J. Nat. Prod.* **2007**, *70*, 412-416. [CrossRef]
95. D'Ambrosio, M.; Guerriero, A.; Debitus, C.; Pietra, F., Leucascandrolide A, a new type of macrolide: the first powerfully bioactive metabolite of calcareous sponges (*Leucascandra caveolata*, a new genus from the coral sea). *Helv. Chim. Acta.* **1996**, *79*, 51-60. [CrossRef]
96. Oku, N.; Takada, K.; Fuller, R.W.; Wilson, J.A.; Peach, M.L.; Pannell, L.K.; McMahon, J.B.; Gustafson, K.R., Isolation, structural elucidation, and absolute stereochemistry of enigmazole A, a cytotoxic phosphomacrolide from the Papua new guinea marine sponge *Cinachyrella enigmatica*. *J. Am. Chem. Soc.* **2010**, *132*, 10278–10285. [CrossRef]
97. Kato, Y.; Fusetani, N.; Matsunaga, S.; Hashimoto, K.; Fujita, S.; Furuya, T., Calyculin A, a novel antitumor metabolite from the marine sponge *Discodermia calyx*. *J. Am. Chem. Soc.* **1986**, *108*, (10), 2780-2781. [CrossRef]
98. Matsunaga, S.; Wakimoto, T.; Fusetani, N., Isolation of four new calyculins from the marine sponge *Discodermia calyx*. *J. Org. Chem.*, **1997**, *62*, (26), 9388-9388. [CrossRef]
99. Yuko, K.; Nobuhiro, F.; Shigeki, M.; Kanehisa, H., Isolation and structure elucidation of calyculins B, C, and D, novel antitumor metabolites, from the marine sponge *Discodermia calyx*. *J. Org. Chem.* **1988**, *53*, 3930-3932. [CrossRef]
100. Matsunaga, S.; Fujiki, H.; Sakata, D.; Fusetani, N., Calyculins E, F, G, and H, additional inhibitors of protein phosphatases 1 and 2A, from the marine sponge *Discodermia calyx*. *Tetrahedron* **2010**, *22*, 2999-3006. [CrossRef]
101. Matsunaga, S.; Wakimoto, T.; Fusetani, N.; Suganuma, M., Isolation of dephosphonocalyculin a from the marine sponge, *Discodermia calyx*. *Tetrahedron Lett.*, **1997**, *38*, 3763-3764. [CrossRef]
102. Dumdei, E.J.; Blunt, J.W.; Munro, M.H.; Pannell, L.K., Isolation of calyculins, calyculinamides, and swinholide H from the New Zealand deep-water marine sponge *Lamellomorpha strongylata*. *J. Org. Chem.*, **1997**, *62*, (8), 2636-2639. [CrossRef]
103. Fu, X.; Schmitz, F.J.; Kelly-Borges, M.; McCready, T.L.; Holmes, C.F.B., Clavosines A-C from the marine sponge *Myriastra clavosa*: potent cytotoxins and inhibitors of protein phosphatases 1 and 2A. *J. Org. Chem.* **1998**, *63*, 7957-7963. [CrossRef]
104. Kehraus, S.; Konig, G.M.; Wright, A.D., A new cytotoxic calyculinamide derivative, geometricin A, from the Australian sponge *Luffariella geometrica*. *J. Nat. Prod.* **2002**, *65*, 1056-1058. [CrossRef]
105. Edrada, R.A.; Ebel, R.; Supriyono, A.; Wray, V.; Schupp, P.; Steube, K.; van Soest, R.; Proksch, P., Swinhoeiamide A, a new highly active calyculin derivative from the marine sponge *Theonella swinhoei*. *J. Nat. Prod.*, **2002**, *65*, 1168-1172. [CrossRef]
106. Wu, Q.H.; Zhang, G.Y.; Wang, B.X.; Li, X.; Yue, S.P.; Chen, J.W.; Zhang, H.W.; Wang, H., Production and identification of inthomycin B produced by a deep-sea sediment-derived *Streptomyces* sp. YB104 based on cultivation-dependent approach. *Curr. Microbio.* **2018**, *75*, 942-951. [CrossRef]

107. Ichiba, T.; Yoshida, W.Y.; Scheuer, P.J.; Higa, T.; Gravalos, D.G., Hennoxazoles, bioactive bisoxazoles from a marine sponge. *J. Am. Chem. Soc.*, **1991**, *113*, (8), 3173-3174. [CrossRef]
108. Higa, T.; Tanaka, J.I.; Kitamura, A.; Koyama, T.; Takahashia, M.; Uchida, T., Bioactive compounds from marine sponges. *Pure. Appl. Chem.* **1994**, *66*, 2227-2230. [CrossRef]
109. Takaishi, S.; Tuchiya, N.; Sato, A.; Negishi, T.; Takamatsu, Y.; Matsushita, Y.; Watanabe, T.; Iijima, Y.; Haruyama, H.; Kinoshita, T.; Tanaka, M.; Kodama, K., B-90063, a novel endothelin converting enzyme inhibitor isolated from a new marine bacterium, *Blastobacter* sp. SANK 71894. *J. Antibiot* **1998**, *51*, 805-815. [CrossRef]
110. Fernandez, R.; Dherbomez, M.; Letourneux, Y.; Nabil, M.; Verbist, J.F.; Biard, J.F., Antifungal metabolites from the marine sponge *Pachastrissa* sp.: new bengamide and bengazole derivatives. *J. Nat. Prod.* **1999**, *62*, 678-680. [CrossRef]
111. Adamczeski, M.; Quinoa, E.; Crews, P., Unusual anthelminthic oxazoles from a marine sponge. *J. Am. Chem. Soc.*, **1988**, *110*, (5), 1598-1602. [CrossRef]
112. Searle, P.A.; Richter, R.K.; Molinski, T.F., Bengazoles C-G from the sponge *Jaspis* sp. Synthesis of the side chain and determination of absolute configuration. *J. Org. Chem.* **1996**, *61*, 4073-4079. [CrossRef]
113. Molinski, T.F., Developments in marine natural products. Receptor-specific bioactive compounds. *J. Nat. Prod.*, **1993**, *56*, (1), 1-8. [CrossRef]
114. Groweiss, A.; Newcomer, J.J.; O'Keefe, B.R.; Blackman, A.; Boyd, M.R., Cytotoxic metabolites from an Australian collection of the sponge *Jaspis* species. *J. Nat. Prod.* **1999**, *62*, 1691-1693. [CrossRef]
115. Rodríguez, J.; Nieto, R.M.; Crews, P., New structures and bioactivity patterns of bengazole alkaloids from a *Choristid* marine sponge. *J. Nat. Prod.* **1993**, *56*, 2034-2040. [CrossRef]
116. Rudi, A.; Kashman, Y.; Benayahu, Y.; Schleyer, M., Amino acid derivatives from the marine sponge *Jaspis digonoxea*. *J. Nat. Prod.* **1994**, *57*, 829-832. [CrossRef]
117. Daletos, G.; de Voogd, N.J.; Muller, W.E.; Wray, V.; Lin, W.; Feger, D.; Kubbutat, M.; Aly, A.H.; Proksch, P., Cytotoxic and protein kinase inhibiting nakijiquinones and nakijiquinols from the sponge *Dactylospongia metachromia*. *J. Nat. Prod.* **2014**, *77*, 218-226. [CrossRef]
118. [118] Hwang, I.H.; Oh, J.; Zhou, W.; Park, S.; Kim, J.H.; Chittiboyina, A.G.; Ferreira, D.; Song, G.Y.; Oh, S.; Na, M.; Hamann, M.T., Cytotoxic activity of rearranged drimane meroterpenoids against colon cancer cells via down-regulation of β-catenin expression. *J. Nat. Prod.* **2015**, *78*, 453-461. [CrossRef]
119. Kobayashi, J.i.; Madono, T.; Shigemori, H., Nakijinol, a novel sesquiterpenoid containing a benzoxazole ring from an Okinawan sponge. *Tetrahedron Lett.* **1995**, *36*, 5589-5590. [CrossRef]
120. Simon P. B. Ovenden; Jonathan L. Nielson; Catherine H. Liptrot; Richard H. Willis; Dianne M. Tapiolas; Anthony D. Wright; Motti, C.A., Sesquiterpene benzoxazoles and sesquiterpene quinones from the marine sponge *Dactylospongia elegans*. *J. Nat. Prod.* **2011**, *74*, 65-68. [CrossRef]
121. Wang, J.; Mu, F.R.; Jiao, W.H.; Huang, J.; Hong, L.L.; Yang, F.; Xu, Y.; Wang, S.P.; Sun, F.; Lin, H.W., Meroterpenoids with protein tyrosine phosphatase 1B inhibitory activity from a *Hyrtios* sp. marine sponge. *J. Nat. Prod.* **2017**, *80*, 2509-2514.[CrossRef]
122. Sun, M.; Zhang, X.; Hao, H.; Li, W.; Lu, C., Nocarbenzoxazoles A-G, benzoxazoles produced by halophilic *Nocardiopsis lucentensis* DSM 44048. *J. Nat. Prod.* **2015**, *78*, 2123-2127. [CrossRef]

123. Venkateswarlu, Y.; Srinivasa Reddy, N.; Ramesh, P.; Venkateswara Rao, J., Coixol a bioactive principle from a marine sponge *Oceanapia* sp. *Biocheml. Syst. Ecol.* **1999**, *27*, 519-520. [CrossRef]
124. Rodríguez, I.I.; Rodríguez, A.D., Homopseudopteroxazole, a new antimycobacterial diterpene alkaloid from P*seudopterogorgia elisabethae*. *J. Nat. Prod,* **2003**, *66*, 855-857. [CrossRef]
125. Rodriguez;, A.D.; Ramırez;, C.; Rodriguez;, I.I.; Gonzalez;, E., Novel antimycobacterial benzoxazole alkaloids, from the west Indian sea whip *Pseudopterogorgia elisabethae*. *Org. Lett.* **1999**, *1*, 527-530. [CrossRef]
126. Rodríguez, I.I.; Rodríguez, A.D.; Wang, Y.; Franzblau, S.G., Ileabethoxazole: a novel benzoxazole alkaloid with antimycobacterial activity. *Tetrahedron Lett.* **2006**, *47*, 3229-3232. [CrossRef]
127. Genta-Jouve, G.; Francezon, N.; Puissant, A.; Auberger, P.; Vacelet, J.; Perez, T.; Fontana, A.; Mourabit, A.A.; Thomas, O.P., Structure elucidation of the new citharoxazole from the Mediterranean deep-sea sponge *Latrunculia* (*Biannulata*) *citharistae*. *Magn. Reson. Chem. Lett.* **2011**, *49*, 533-536. [CrossRef]
128. Julianti, E.; Lee, J.H.; Liao, L.; Park, W.; Park, S.; Oh, D.C.; Oh, K.B.; Shin, J., New polyaromatic metabolites from a marine-derived fungus *Penicillium* sp. *Org. Lett.* **2013**, *15*, 1286-1289. [CrossRef]
129. Takahashi, Y.; Kubota, T.; Shibazaki, A.; Gonoi, T.; Fromont, J.; Kobayashi, J., Nakijinamines C-E, new heteroaromatic alkaloids from the sponge *Suberites* species. *Org. Lett.* **2011**, *13*, 3016-3019. [CrossRef]
130. Hohmann, C.; Schneider, K.; Bruntner, C.; Irran, E.; Nicholson, G.; Bull, A.T.; Jones, A.L.; Brown, R.; Stach, J.E.; Goodfellow, M.; Beil, W.; Kramer, M.; Imhoff, J.F.; Sussmuth, R.D.; Fiedler, H.P., Caboxamycin, a new antibiotic of the benzoxazole family produced by the deep-sea strain *Streptomyces* sp. NTK 937. *J. Antibiot.* **2009**, *62*, 99-104. [CrossRef]
131. Dattelbaum, J.D.; Singh, A.J.; Field, J.J.; Miller, J.H.; Northcote, P.T., The nitrogenous hamigerans: unusual amino acid-derivatized aromatic diterpenoid metabolites from the New Zealand marine sponge *Hamigera tarangaensis*. *J. Org. Chem.* **2015**, *80*, 304-312. [CrossRef]
132. Liu, L.L.; Xu, Y.; Han, Z.; Li, Y.X.; Lu, L.; Lai, P.Y.; Zhong, J.L.; Guo, X.R.; Zhang, X.X.; Qian, P.Y., Four new antibacterial xanthones from the marine-derived actinomycetes *Streptomyces caelestis*. *Mar. Drugs* **2012**, *10*, 2571-2583. [CrossRef]
133. Wakimoto, T.; Tan, K.C.; Abe, I., Ergot alkaloid from the sea slug *Pleurobranchus forskalii*. *Toxicon* **2013**, *72*, 1-4. [CrossRef]
